# A New Fiji-Based Algorithm That Systematically Quantifies Nine Synaptic Parameters Provides Insights into *Drosophila* NMJ Morphometry

**DOI:** 10.1371/journal.pcbi.1004823

**Published:** 2016-03-21

**Authors:** Bonnie Nijhof, Anna Castells-Nobau, Louis Wolf, Jolanda M. Scheffer-de Gooyert, Ignacio Monedero, Laura Torroja, Lluis Coromina, Jeroen A. W. M. van der Laak, Annette Schenck

**Affiliations:** 1 Department of Human Genetics, Donders Institute for Brain, Cognition and Behaviour, Radboud University Medical Center, Nijmegen, the Netherlands; 2 Microscopical Imaging Centre (MIC), Radboud University Medical Center, Nijmegen, the Netherlands; 3 Department of Biology, Universidad Autónoma de Madrid, Madrid, Spain; 4 Department of Clinical and Experimental Medicine, Linköping University, Linköping, Sweden; 5 Research Group on Statistics, Econometrics and Health (GRECS) and CIBER of Epidemiology and Public Health (CIBERESP), University of Girona, Girona, Spain; 6 Department of Pathology, Radboud University Medical Center, Nijmegen, the Netherlands; Université Paris Descartes, Centre National de la Recherche Scientifique, FRANCE

## Abstract

The morphology of synapses is of central interest in neuroscience because of the intimate relation with synaptic efficacy. Two decades of gene manipulation studies in different animal models have revealed a repertoire of molecules that contribute to synapse development. However, since such studies often assessed only one, or at best a few, morphological features at a given synapse, it remained unaddressed how different structural aspects relate to one another. Furthermore, such focused and sometimes only qualitative approaches likely left many of the more subtle players unnoticed. Here, we present the image analysis algorithm ‘Drosophila_NMJ_Morphometrics’, available as a Fiji-compatible macro, for quantitative, accurate and objective synapse morphometry of the Drosophila larval neuromuscular junction (NMJ), a well-established glutamatergic model synapse. We developed this methodology for semi-automated multiparametric analyses of NMJ terminals immunolabeled for the commonly used markers Dlg1 and Brp and showed that it also works for Hrp, Csp and Syt. We demonstrate that gender, genetic background and identity of abdominal body segment consistently and significantly contribute to variability in our data, suggesting that controlling for these parameters is important to minimize variability in quantitative analyses. Correlation and principal component analyses (PCA) were performed to investigate which morphometric parameters are inter-dependent and which ones are regulated rather independently. Based on nine acquired parameters, we identified five morphometric groups: NMJ size, geometry, muscle size, number of NMJ islands and number of active zones. Based on our finding that the parameters of the first two principal components hardly correlated with each other, we suggest that different molecular processes underlie these two morphometric groups. Our study sets the stage for systems morphometry approaches at the well-studied Drosophila NMJ.

## Author Summary

Altered synapse function underlies cognitive disorders such as intellectual disability, autism and schizophrenia. The morphology of synapses is crucial for their function but is often described using only a small number of parameters or categories. As a consequence, it is still unknown how different aspects of synapse morphology relate to each other and whether they respond in a coordinated or independent manner. Here, we report a sensitive and multiparametric method for systematic synapse morphometry at the *Drosophila* Neuromuscular Junction (NMJ), a popular model for mammalian synapse biology. Surveying a large NMJ image repository, we provide insights in the natural variation of NMJ morphology as a result of differences in gender, genetic background and abdominal body segment. We show which synapse parameters correlate and find that parameters fall into five groups. Based on our findings, we propose that two of them, NMJ size and geometry, are controlled by different molecular mechanisms. Our study provides insights into the design principles of a model synapse and tools that can be applied in future studies to identify genes that modulate or co-orchestrate different aspects of synapse morphology.

## Introduction

Normal brain function relies on functional neuronal networks in which neurons connect and communicate with one another. Communication primarily takes place at chemical synapses, where neurotransmitters are released from the presynaptic compartment of a neuron and activate receptors at the postsynaptic compartment of its target cell. Abnormal synaptic development and function have been found to underlie cognitive disorders such as intellectual disability, autism spectrum disorder and schizophrenia [1–5]. The morphology and function of synapses are highly intertwined [6–9] and morphological aspects have therefore been studied extensively to gain further insight into the regulatory networks underlying synaptic function. Mammalian dendritic spines change shape upon maturation and plasticity from long, thin filopodia-like structures to typical stubby and mushroom-shaped postsynaptic compartments of increased efficacy [10,11]. In Drosophila, synaptic structure and activity is modulated according to circadian timing [12–15] or upon experienced-dependent or stimulated activity [16,17], to name only three examples.

Despite the central interest in synapse morphology in neuroscience -studied at different developmental stages, upon genetic or environmental perturbation and in different organisms- it is still largely unknown how different structural aspects relate to one another and adapt in a coordinated manner when changes are induced. Systematic synapse morphometry could shed light on these poorly understood relationships. In genetically unperturbed conditions, such insights would be crucial to understand the developmental design principles that shape the synapse. This in turn would provide a basis to identify the genetic players that drive the required coordinated structural changes during synaptic development and plasticity with higher sensitivity.

As an initial step into quantitative, correlative synapse morphometry, we have turned to an identifiable, methodologically and genetically accessible synaptic terminal: the Drosophila larval neuromuscular junction (NMJ). The Drosophila NMJ is an extensively studied and well-established in vivo model for glutamatergic synapse biology [18,19]. The synaptic terminal, a branched chain of synaptic boutons, is formed by the motor neuron and gets surrounded by the subsynaptic reticulum (SSR) as it invades its target muscle [20]. Boutons are periodic enlargements [21] that host the presynaptic release sites, ‘active zones’ [20], at which the synaptic vesicles dock to the presynaptic membrane to release their neurotransmitters. Together with the exactly opposed postsynaptic receptor complex, the active zone forms the chemical synapse [20]. Large scale genetic screens at the NMJ have been very successful in identifying genes and molecular mechanisms of synapse development [22–31]. However, so far, these screens have largely relied on visual inspection and semi-quantitative scoring of a limited amount of morphometric features. While this has uncovered main determinants of NMJ morphology, it is likely that the extent of the regulatory networks remained undiscovered.

In this study, we developed a macro in Fiji (an open-source image analysis software [32]) to quantitatively assess nine morphometric features in a large number of glutamatergic NMJs based on high-content fluorescence microscopy images. We found the macro to accurately assess eight of them (NMJ area, perimeter, total length, longest branch length, number of islands, number of branches and branching points and number of active zones), making it suitable for high-throughput analyses of synapse morphology. Here, in preparation for reverse genetic approaches, this method was applied to two isogenic host strains of genomewide RNAi libraries (VDRC [33]; see Methods) to build large wt-like control datasets. Using these data, we followed a systems biology approach by using the differences in gender, abdominal body segment and genetic background as natural sources of biological variation to gain insights into the (in)dependencies and correlations of the measured morphometric NMJ features. Our study is the first to investigate the systems properties of the well-studied Drosophila NMJ, providing new insights into the design principles of a synapse.

## Results

### Generating a large NMJ image repository

We generated a large collection of NMJ images in two different genotypes, the isogenic host strain for the GD and KK RNAi libraries of the Vienna Drosophila Resource Center [33] crossed to a panneuronal elav promotor line (see Methods). The obtained larvae were dissected and stained with antibodies against two key components of the NMJ, the discs large 1 protein (Dlg1 –the ortholog of mammalian PSD-95) and bruchpilot (Brp—sole ortholog of human ELKS/CAST/ERC proteins [34]), to visualize general synapse morphology and active zones [35], respectively. We focused on abdominal segments A2-A5, which are best accessible in larval ‘open book’ preparations. In total we acquired microscopic images of 1576 NMJs in 397 larvae.

### A Fiji macro for high-throughput, objective and multiparametric NMJ analysis

It is a laborious undertaking to measure NMJ features (semi) manually, especially when several NMJ features are of interest. To support high-throughput analyses and achieve objective quantification, we set out to develop a macro for computer-assisted morphometry that can accurately quantify high-content, non-confocal images.

The macro ‘Drosophila_NMJ_morphometrics’, was developed using the open source Fiji platform [32] and is made available via figshare, a public repository where users can make their research outputs available: https://figshare.com/s/ec634918c027f62f7f2a [36]. For usage, Fiji needs to be installed and the macro has to be downloaded and saved with the extension (.ijm) into the Fiji Plugins folder. The macro will appear in the Plugins menu under the name Drosophila_NMJ_Morphometrics. Upon running the macro a graphical interface displays the default settings of the macro, which can be adjusted according to the customer’s needs. The ‘help’ option offers additional information. A point-by-point protocol for using the macro is provided in the supplementary material (S1 Protocol).

The macro consists of three sub macro’s that can be used separately or run in a consecutive manner to analyze and process images (via checkbox options of the macro interface). The first sub macro “Convert to stack” identifies all image files available and creates stacks and maximum intensity projections of both channels. The second sub macro “Define ROI” presents the projections to manually delineate the region of interest (ROI). As we were interested in type 1b NMJs on muscle 4, this manual step was required to exclude type 1s synaptic terminals on the same muscle, and occasionally exclude synapses on nearby muscles that are present in the images. The third sub macro “Analyze” applies fully automated analysis through all stacks within the limits of the ROI. For each NMJ, nine morphological parameters are measured (described in more detail below) and processed to an (.txt) output file. Images are processed to a result picture, in which the delineation of the automatically recognized NMJ features is presented.

**Fig 1:**
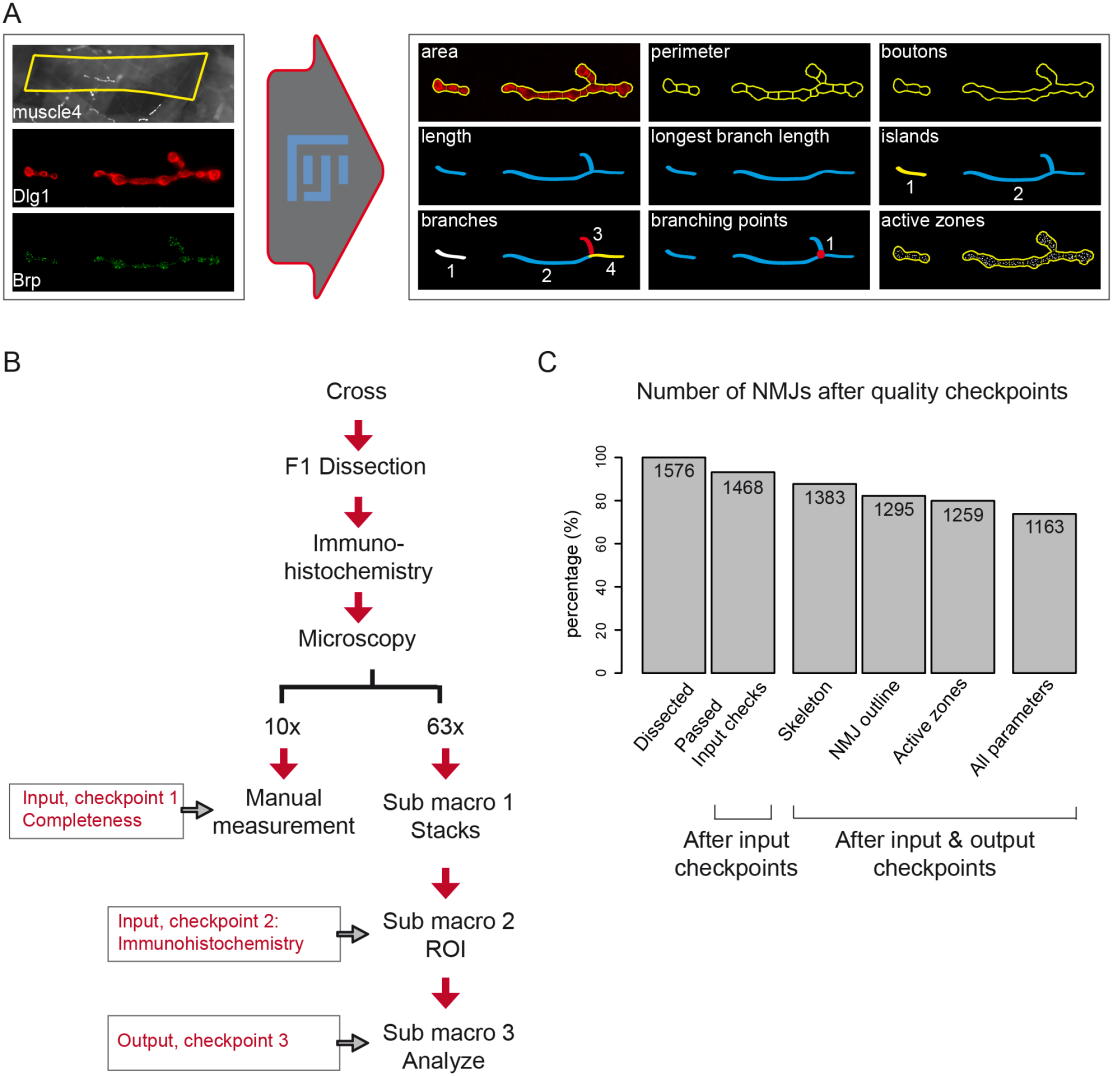
A Fiji macro to systematically quantify NMJ features. Representation of the Fiji-based macro for NMJ systems analysis (A). The macro was developed using Dlg1 and Brp image stacks as input (left to the arrow). For each NMJ, the indicated nine NMJ features are quantified. The NMJ area, perimeter and boutons are deduced from the NMJ outline and inner segmentations (indicated in yellow). The skeleton (blue) provides the five features total length, longest branch length (sub branches are excluded), the number of Dlg1-based islands (unconnected Dlg1 compartments, in blue and yellow), the number of branches (in different colors), the number of branching points (red dot). The Brp channel is used to count the number of active zones within the NMJ outline (white dots within the yellow outline). The NMJ muscle area is measured at lower magnification by the Fiji freehand selection (yellow box rectangle). NMJs are oriented anterior left, dorsal up. Schematic representation of the NMJ workflow (B). Larvae are dissected and NMJs are stained for Dlg1 and Brp and captured in images at the two magnifications 10x and 63x. Lower magnification snapshots are used to measure the muscle4 area and are simultaneously checked to ensure that the full NMJ is captured at high magnification (= checkpoint 1). Images at high magnification are progressed to stacks and projections in which the region of interest (ROI) is defined to exclusively analyze type 1b NMJs. Images are excluded in checkpoint 2 if the specificity of type 1b cannot be guaranteed or if the quality of the immunohistochemistry is poor. The first two bars of the bar graph (C) represent the amount of NMJs and the percentage that passed the two input checkpoints (93.15%). Sub macro 3 processes the NMJ towards quantitative output and the macro-annotated images are evaluated (= checkpoint 3). Bars three to five represent the percentage of NMJs per threshold that passed the output quality checkpoint (87.75% for the NMJ skeleton; 82.17% for the NMJ outline and 79.89% for Active zones). The final bar represents the percentage of investigated NMJs for which all features were of high quality (73.79%).

During image analysis, from each NMJ three structures were derived: 1) NMJ outline, 2) NMJ skeleton and 3) number of Brp-positive active zones. Technical details underlying each derived structure are described in the Methods section Image Analysis. The NMJ outline is used to determine the NMJ area and its perimeter and a subsequent watershed separation provides the number of boutons (Fig 1A, NMJ outline indicated in yellow). From the skeleton (Fig 1A, indicated in blue) five NMJ features are deduced: the total NMJ length, the sum of the length of the longest continuous path connecting any two end points (longest branch length), the number of unconnected Dlg1-stained compartments per NMJ (referred to as ‘islands’), the number of branches and the number of branching points (one branching point connects three or more branches). The number of active zones was determined by counting Brp-positive spots in the Brp-channel (Fig 1A, indicated by white foci). Taken together, the macro determines three derivatives per NMJ, from which it deduces nine morphological NMJ features. Eight of the nine features are based on the Dlg1- and one on the Brp-channel. Fig 1A provides a schematic overview of all nine NMJ features.

### Image segmentation

The Fiji macro was used to process our dataset of 1576 NMJ images. We checked the quality of our images at low magnification (Fig 1B, input checkpoint 1) and found that for 33 images (2.09%) the NMJ was not fully captured in the acquired stack. Furthermore, a second checkpoint at high magnification (Fig 1B, input checkpoint 2) revealed 35 images (2.22%) in which the NMJ was partially out-of-focus, 25 images (1.59%) with weak staining and 21 images (1.33%) from which we could not guarantee the specificity of type 1b. For the remaining 1468 NMJ images (93.15%) (Fig 1C), the macro-annotated images were used to evaluate the obtained NMJ outline, skeleton and Brp-positive active zones per NMJ image (Fig 1B, output checkpoint 3). Variability in staining intensity led to a relatively high amount of images from which part of the NMJ outline was not recognized (n = 90, 6.13% from 1576), certain areas that lack Brp-positive active zones (n = 121, 8.24%) or a combination of these two events (n = 95, 6.47% from 1576). Images with skeleton misannotations (n = 186, 12.67% from 1576) were manually corrected. In summary, after three rounds of quality checks, we remained with a NMJ dataset of 1295 images for the NMJ-outline features (82.17% from initial; 86.62% from 1468), 1383 images for the NMJ-skeleton features (87.75% from initial; 92.51% from 1468) and 1259 images for the active zones (79.89% from initial; 84.21% from 1468). In total, we obtained 1163 NMJ images from which all features past the three quality rounds (73.79% from initial, 79.22% from 1468).

### Macro validation

**Fig 2:**
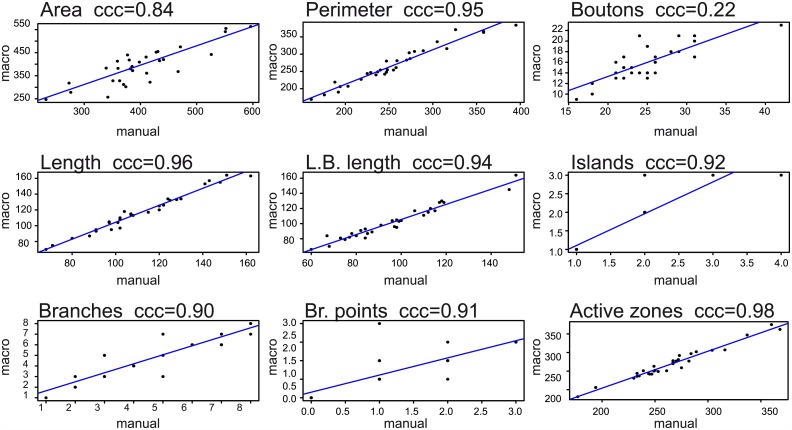
Macro validation. The graphs represent the concordance correlation coefficient (ccc) between manually assessed NMJs on the x-axis and the macro results on the y-axis for each feature separately. Each data point represents the intersection of macro and manual measurement for a given NMJ sample (n = 30). Because of identical (whole number) counts, data points are often superimposed for islands, branches and branching points.

In absence of truly objective NMJ measures, we compared the results obtained with the macro to the manual counts of two experienced experimenters for 30 NMJ images (S1 Table). We first investigated the sample distributions to determine the deviation between manual and macro assessment over the complete set of images. The 95% confidence intervals largely overlapped with each other for the parameters NMJ area, perimeter, length, longest branch length, islands, branches, branching points, and active zones (S1 Fig). Thus, no significant differences were found between the distributions of macro and manual assessment for these NMJ features (Table 1). However, the macro resulted in a significantly lower amount of bouton counts (macro: 16 boutons per NMJ; manual; 25 per NMJ; p<0.0001) (Table 1). The 95% confidence interval widths were highly comparable between macro and manual counts, indicating that the macro does not add additional noise to the outcome (S1 Fig). Secondly, we investigated the deviation between manual and macro evaluation per given sample, expressed as %deviation or sensitivity and specificity. The %deviation per given sample was often negative for the NMJ perimeter, length and longest branch length (S1 Table), indicating that the macro measures somewhat higher absolute values as compared to the manual counts. On average, the boutons showed a six times higher % deviation between macro and manual counts compared to the NMJ area, perimeter, length and longest branch length (Table 1). Sensitivity (the proportion of positive results that is indeed a true positive) and specificity (the proportion of true positives that is identified as such) was determined per NMJ image for the discrete NMJ features islands, branches, branching points and active zones (S1 Table). On average, all four parameters scored >91% on sensitivity and >92% on specificity (Table 1). Finally, Lin’s concordance correlation coefficient (ccc), which describes the reproducibility between two evaluation methods, was calculated to determine the deviation of the acquired macro data from the perfect concordance (x = y) (Table 1, Fig 2) [37]. On a scale from 0.00 to 1.00, the macro scored ccc’s ≥0.84 for all NMJ features but bouton count. Bouton count resulted in a ccc of 0.22 (C.I.95% 0.10–0.32), which indicates that macro and manual performance are discordant. In summary, the macro assessed nine NMJ features, eight of which were successfully validated with high concordance correlations. We therefore mainly focused on these eight features in all subsequent analyses.

**Table 1:** Macro validation on high-content microscopy images.

NMJ feature	Average manual count	Macro count	Distribution (p-value)	Ccc	Average deviation	Sensitivity	Specificity
***NMJ area***	403μm^2^	392μm^2^	0.60	0.84	2.10%	x	x
***Perimeter***	257μm	269μm	0.38	0.95	5.10%	x	x
***Boutons***	25	16	<0.0001	0.22	35.02%		
***NMJ Length***	111μm	117μm	0.41	0.96	4.55%	x	x
***Longest branch length***	96μm	102μm	0.34	0.94	6.11%	x	x
***Islands***	1.73	1.73	0.91	0.92	x	98.89%	99.17%
***Branches***	4.47	4.60	0.77	0.90	x	93.94%	97.30%
***Branching points***	1.33	1.53	0.49	0.91	x	91.39%	98.33%
***Active zones***	266	269	0.73	0.98	x	91.45%	92.64%

**Fig 3:**
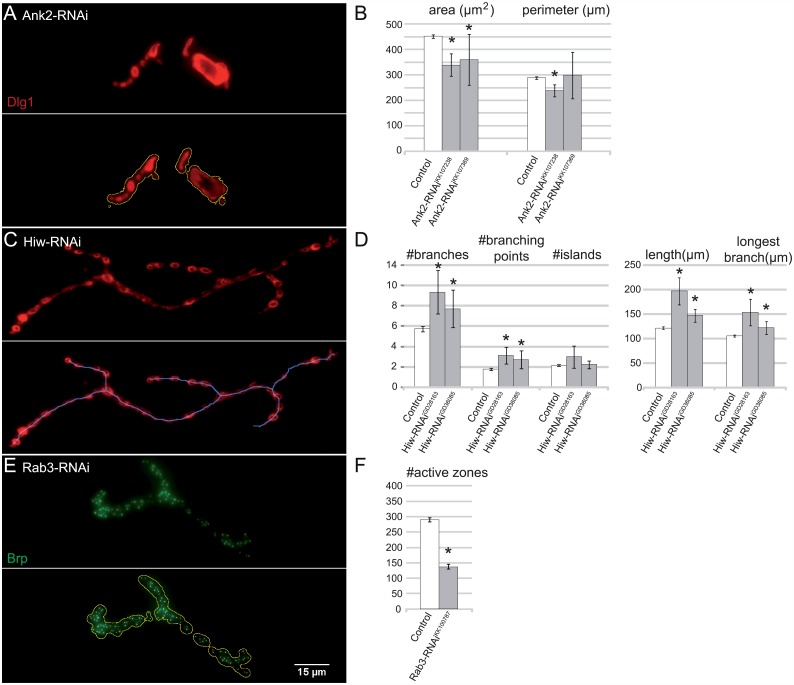
Evaluation of reproducibility. Macro assessment and quantification of NMJs on muscle 4. Ankyrin2 knockdown (*w;UAS-Dicer-2/UAS-KK107238;elav-Gal4/+*, n = 18 and *w;UAS-Dicer-2/UAS-KK107369;elav-Gal4/+*, n = 19) was confirmed to result in smaller NMJ size compared to genetic background controls (*w;UAS-Dicer-2/+;elav-Gal4/+*, n = 500) when using the macro (A-B). The macro-annotated outline is indicated in yellow. Highwire knockdown (*w;UAS-Dicer-2/UAS-GD28163;elav-Gal4/+*, n = 15 and *w;UAS-Dicer-2/UAS-GD36085;elav-Gal4/+*, n = 26) resulted in longer and more branched NMJs compared to genetic background control NMJs (*w;UAS-Dicer-2/+;elav-Gal4/+*, n = 402) (C-D). The macro-annotated skeleton is indicated in blue. Rab3 knockdown (*w;UAS-Dicer-2/UAS-KK100787;elav-Gal4/+*, n = 18) resulted in NMJs with a lower number of Brp-positive active zones compared to genetic background controls (*w;UAS-Dicer-2/+;elav-Gal4/+*, n = 476) (E-F). The macro-annotated Brp-positive active zones are indicated as white foci. Error bars indicate 95% confidence interval and asterisks represent significance (p_adj_ < 0.05).

To further validate the macro, we tested the reproducibility of published findings on mutants with altered synaptic parameters for each of the three principal image segmentation procedures performed by our macro (NMJ outline, skeleton and active zones). We and others have shown that Ankyrin 2 (*Ank2*, CG42734) mutant [38,39] or knockdown [40] flies present with fused boutons and smaller NMJs. Here, we used panneuronal Ank2 knockdown NMJs as a positive control to validate the macro’s NMJ outline (Fig 3A and 3B). The NMJ area was significantly smaller upon Ank2 knockdown by two independent RNAi strains (Ank2-RNAi^KK107238^ 339μm^2^, p_adj_ = 2.18E-08; Ank2-RNAi^KK107369^ 361μm^2^, p_adj_ = 1.20E-05), compared to our genetic background control dataset (452 μm^2^). The NMJ perimeter was only significantly smaller for the stronger RNAi strain (control 289μm; Ank2-RNAi^KK107238^ 238μm, p_adj_ = 1.82E-03). Highwire (*hiw*, CG32592) is a known regulator of NMJ length and the extent of branching and mutants typically present with long, highly branched NMJs [41]. Our macro reproduced the mutant phenotype in NMJs that have a panneuronal knockdown of Highwire, again by using two independent RNAi strains (Fig 3C and 3D). The NMJ skeleton-derived parameters length (Hiw-RNAi^GD28163^ 197μm, p_adj_ = 3.10E-25; Hiw-RNAi^GD36085^ 147μm, p_adj_ = 7.31E-07; control 122μm), longest branch length (Hiw-RNAi^GD28163^ 154μm, p_adj_ = 2.02E-13; Hiw-RNAi^GD36085^ 122μm, p_adj_ = 4.62E-04; control 106μm), number of branches (Hiw-RNAi^GD28163^ 9.33, p_adj_ = 2.10E-04; Hiw-RNAi^GD36085^ 7.69, p_adj_ = 2.52E-02; control 5.74) and number of branching points (Hiw-RNAi^GD28163^ 3.13, p_adj_ = 6.74–04; Hiw-RNAi^GD36085^ 2.73, p_adj_ = 3.31E-02; control 1.79) are all significantly higher (120–180%) compared to the genetic background control dataset. Lastly, the GTPase Rab3 is required for proper bruchpilot distribution and the mutant (*rup*) presents with a reduced number of Brp-positive active zones (81 compared to 298 in control NMJs on muscle 4) [42]. The macro reproduced this phenotype upon panneuronal knockdown (Rab3-RNAi^KK100787^ 138 Brp-positive active zones; control 290 Brp-positive active zones; p = 4.43E-29) (Fig 3E and 3F).

### A quantitative NMJ dataset

**Fig 4:**
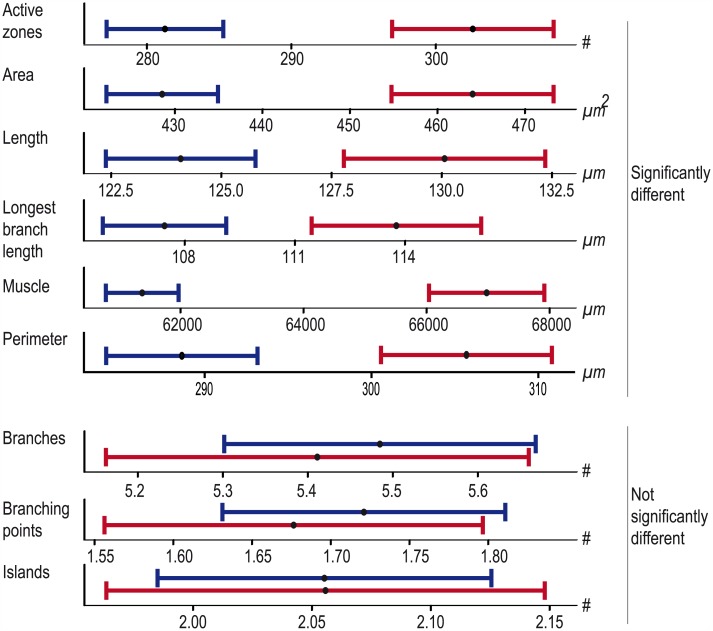
The influence of gender on NMJ features. The plots display the mean value (black dot) and 95% confidence interval for each indicated feature. Males are represented in blue, females in red. The features are significantly different between sexes for the size-related NMJ features (upper panel), whereas they largely overlap for structural NMJ features (lower panel).

The large collection of objectively quantified NMJ data offered the possibility to look at systematic differences in NMJ morphometry for gender, genetic background and body segment. For this purpose we restricted the dataset to images from which we obtained data for all nine features, including muscle measurements (due to the latter requirement an additional 62 NMJ images were excluded). We divided the dataset into male- (n = 724) and female-specific (n = 377) data and evaluated the differences between both sexes. We found that six features significantly differed from each other (p_adj_ < 0.05), with males showing lower average values than females: active zones (♂ 281; ♀ 303; p_adj_ = 1.51E-08), NMJ area (♂ 429μm^2^; ♀ 464μm^2^; p_adj_ = 8.43E-09), perimeter (♂ 289μm; ♀ 306μm; p_adj_ = 7.26E-06), NMJ total length (♂ 124μm; ♀ 130μm; p_adj_ = 1.65E-04), longest branch length (♂ 107μm; ♀ 114μm; p_adj_ = 8.30E-05) and muscle area (♂ 61377μm^2^; ♀ 66976μm^2^; p_adj_ = 1.98E-15) (Fig 4). In contrast, gender did not significantly impact the number of branches (♂ 5.5; ♀ 5.4; p_adj_ = 1.00), branching points (♂ 1.7; ♀ 1.7; p_adj_ = 1.00) and Islands (♂ 2.1; ♀ 2.1; p_adj_ = 1.00). Taken together, this suggests that the branching geometry is similar for both sexes, whereas size is not.

**Fig 5:**
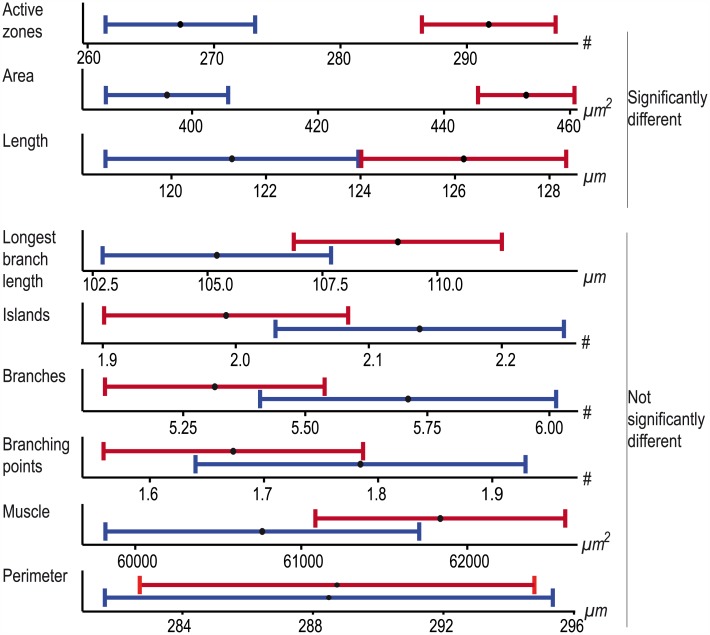
The influence of the genetic background on NMJ features. The plots display the mean value (black dot) and 95% confidence interval for the genetic background of the VDRC GD library (blue) versus the genetic background of the KK library (red) for each feature. The upper panel represents the features that are significantly different between the two genetic backgrounds and the lower panel represents the features for which the genetic background does not significantly influence the outcome.

Next, we aimed to determine the influence that the genetic background might have on our NMJ features, focusing on two genetic backgrounds relevant for large scale reverse genetic screening. We divided our dataset, considering males only, into two genetic backgrounds, deriving from GD (n = 311) versus KK VDRC RNAi libraries (n = 413), and compared these between each other for each NMJ feature (Fig 5). Three of the nine features showed a significant difference between the two tested genetic backgrounds: active zones (GD 267; KK 292; p_adj_ = 2.21E-08), NMJ area (GD 396μm^2^; KK 453μm^2^; p_adj_ = 1.98E-15) and length (GD 121μm; KK 126μm; p_adj_ = 3.78E-02). No significant differences were observed for the other six features: longest branch length (GD 105μm; KK 109μm; p_adj_ = 1.32E-01, Islands (GD 2.1; KK 2.0; p_adj_ = 1.32E-01), branches (GD 5.7; KK 5.3; p_adj_ = 3.96E-01), branching points (GD 1.8; KK 1.7; p_adj_ = 8.38E-01), muscle area (GD 60765μm^2^; KK 61838μm^2^; p_adj_ = 3.29E-01) and NMJ perimeter (GD 288μm; KK 289μm; p_adj_ = 9.57E-01). This data shows that the genetic background can be a significant source of “variance” at the NMJ.

**Fig 6:**
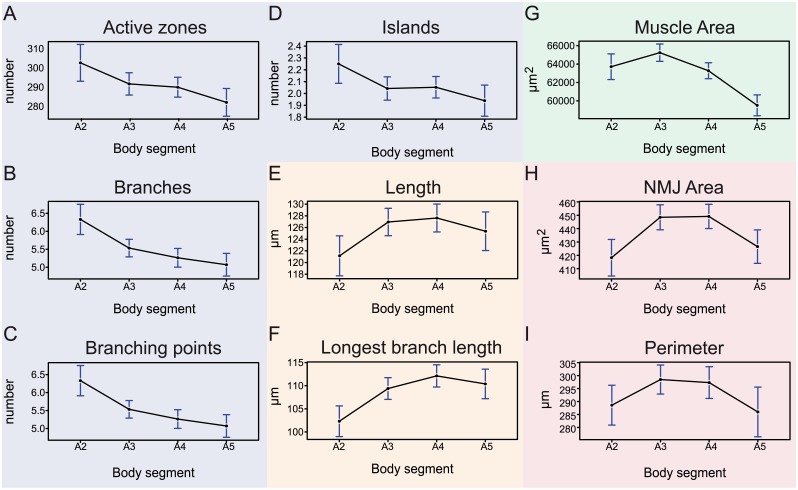
The influence of the abdominal body segments on NMJ features. The plots display the mean (dot) and 95% confidence interval (blue error bars) of the NMJ values for each feature over the abdominal body segments A2 (n = 148), A3 (n = 356), A4 (n = 399) and A5 (n = 198). Four different categories are indicated by color. The blue category includes the features active zones (A), branches (B), branching points (C) and islands (D) and show a relative decrease from anterior to posterior with, except for the islands, A2 being significantly different. The second, orange, category includes the length (E) and longest branch length (F), showing an opposite pattern, in which A2 is significantly smaller than the other segments. The third category (green) includes the muscle area (G), that shows a significant increase in values from A2 to A3 and a significant decrease from A3 to A5. The last category (red) includes the features NMJ area (H) and perimeter (I), which shows a similarity between A2 and A5 versus A3-A4, only significant for NMJ area.

The literature reports data for abdominal body segments in the range of A2-A5, whereby studies report on evaluated NMJ data at one segment or a combination of different segments [19,43]. We aimed to quantitatively determine whether among these segments NMJs show considerable differences in one or several features. Consequently, we divided the dataset into four groups, each representing one segment. We did find differences among features across the 4 evaluated segments, following different spatial patterns. The number of active zones, branches and branching points showed a relative decrease from anterior to posterior (Fig 6A–6C). However, only segment A2 showed significant differences to (some of) the other segments (Table A in S2 Table). The number of islands followed the same pattern, but the values were not significantly different over the different segments (Fig 6D; Table A in S2 Table). The total length and longest branch length showed the opposite pattern; segment A2 NMJs were significantly shorter than NMJs of segments A3-A5 (Fig 6E and 6F; Table A in S2 Table). The muscle area of segment A2 was also significantly smaller compared to segment A3. The size peaks in segment A3 and significantly decreased in the segments A4 and A5. The muscle size of segment A5 was significantly smaller than that observed for A2 (Fig 6G; Table A in S2 Table). The NMJ area formed the fourth category that showed significant increase from segment A2 to A3 and a significant decrease to the same level from A4 to A5. The NMJ perimeter behaved very similar, although values were not significantly different from one another (Fig 6H and 6I; Table A in S2 Table). An overview of the number of cases, mean, confidence intervals en p-values is provided in S2A Table. Although not always significant, consistent patterns were observed in each group of gender and genetic background (Tables B-E in S2 Table). Taken together, the NMJ features could be subdivided in four groups with different patterns over the abdominal segments A2-A5: i) active zones, branches, branching points and islands, ii) length and longest branch length, iii) muscle area and iv) NMJ area and perimeter.

### Principal component analysis of NMJ morphology

**Fig 7:**
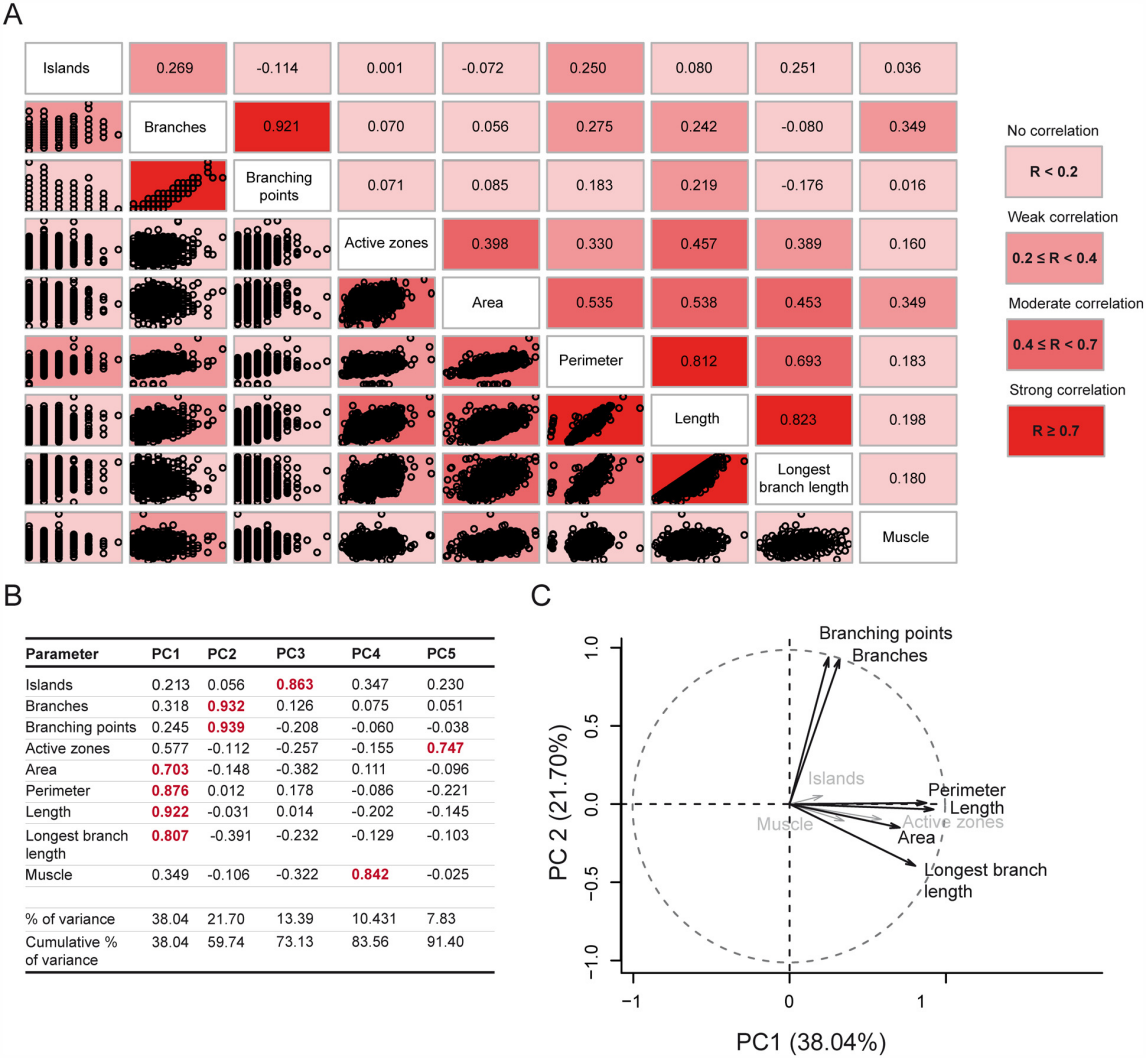
Principal components of NMJ morphometry. The correlation-matrix represents the pair wise correlations between all possible feature pairs, displayed as scatter plots (bottom left side) or as correlation coefficients (top right side) (A). The matrix is ordered so that those with the highest correlation are closer together. Correlation strength is color coded, ranging from light reddish (no-weak correlation) to an intense red color (for strongly correlating features). Five principle components were determined and the contribution of each feature to a certain component is summarized, together with the percentage of variance that is explained by that principal component and the cumulative percentage of total variance (B). The most contributing features per principal component (PC) are indicated in red. The first two components, which together make up for 59.83% of total variance are plotted with the first principal component (PC1) on the x-axis and the second principal component (PC2) on the y-axis (C). The arrow length reflects the contribution of each feature to the first two principal components: features showing a major contribution to PC1 or PC2 are indicated in black, and NMJ features with a minor contribution are grey.

Finally, we used our morphometric dataset to determine which NMJ features might correlate with each other and which features appear comparatively independent, to reveal coordinated aspects of NMJ morphology. A pair wise correlation analysis was performed, in which the correlations of all possible feature pairs were determined and ordered accordingly (Fig 7A). As one might have expected, the strongest positive correlation was found between branches and branching points (R = 0.92), indicating that these features can almost predict each other. The other group of moderately-to-strongly correlating features included the size-related features NMJ area, perimeter, length and longest branch length (0.45<R<0.82). The number of active zones correlated to a lesser extent with this group (0.36<R<0.47). We only observed a weak correlation between the NMJ area and the muscle area (R = 0.35). Both the features muscle area and number of islands seemed to behave as independent features, lacking any moderate (0.4<R≤0.7) or strong (R>0.7) correlation with any of the other NMJ features.

We applied a principal component analysis (PCA), a statistical method to reduce the dimensionality of a dataset, and summarized our data in five different components. This aggregation was the most acceptable because it explained 91% of the variance of our data and classified each of the measured NMJ features on one of these components (Fig 7B; Table A in S3 Table). The size-related features NMJ area, perimeter, length and longest branch length constitute the first principal component, which explained 38.2% of the total variance. The features branches and branching points contributed most to the second principal component (21.7% of the total variance), thus the second component mainly accounted for NMJ geometry. The first two components explained almost 60% of all variance. The angles between the features contributing to the first versus the second principal components were around 90°, indicating that the variables NMJ area, perimeter, length and longest branch length hardly correlated with the variables branches and branching points. This is in agreement with the above reported correlation coefficients (Fig 7B and 7C). The features islands, muscle and active zones contributed most to the third (13.4%), fourth (10.4%) and fifth (7.7%) principal component, respectively. Based on these results, we defined five morphometric groups with a variety of mutual kinship: 1) NMJ size (NMJ area, perimeter, length and longest branch length), 2) geometry (branches and branching points), 3) islands, 4) muscle area and 5) number of active zones. Important to note is that the active zones also showed a moderate correlation and contribution to the NMJ-size features underlying most of the first component. This suggests that the number of active zones is at least partially coordinated with NMJ size. We obtained comparable results when applying PCA on datasets specific to one combination of gender and genetic background library (Tables A-E in S3 Table) or datasets specific for one abdominal body segment (Tables F-I in S3 Table).

In summary, our data showed that synaptic size varies the most within (natural) populations, followed by the branching geometry. It is remarkable that the size- and geometry-related features hardly correlated, suggesting that these features are differentially regulated during larval NMJ development.

### Wider macro applicability

Our macro was designed to cope with the challenges of high-throughput images with limited resolution and quality. However, to ensure wide applicability we also tested our macro on confocal images (S3 Fig). Following a similar strategy as above, manual and macro counts were compared between n = 15 NMJ confocal images, co-labeled for Dlg1 and Brp (Tables A-I in S4 Table). No significant differences and ccc scores ≥0.83 were found for the NMJ features NMJ area, perimeter, length, longest branch length, number of islands, number of branches, number of branching points and number of active zones when manual measurements were compared to the macro assessment (Table 2). We found, however, a significant difference between manual and macro bouton count (p = 0.01; ccc = 0.55), which indeed confirmed that the marker and not the technique is causing this difference. It generally applied that the better the quality of the image, the better the macro performed.

**Table 2:** Macro validation on confocal microscopy images.

NMJ feature	Manual count	Macro count	Significance (p-value)	Ccc	Average deviation	Sensitivity	Specificity
***NMJ area***	356 μm^2^	355 μm^2^	0.98	0.91	0.98%	x	x
***Perimeter***	247 μm	249 μm	0.93	0.92	1.99%	x	x
***Boutons***	22	16	0.01	0.55	27.47%		
***NMJ Length***	108 μm	107 μm	0.96	0.99	0.30%	x	x
***Longest branch length***	99 μm	98 μm	0.96	0.99	0.14%	x	x
***Islands***	1.47	1.33	0.46	0.91	x	100%	93.33%
***Branches***	2.93	3.27	0.95	0.92	x	92.67%	96.00%
***Branching points***	0.67	1.00	0.60	0.83	x	92.78%	93.33%
***Active zones***	250	247	0.88	0.94	x	92.23%	91.26%

We further tested the applicability of our software to other synaptic markers. Horseradish peroxidase (Hrp) is a neuronal membrane marker, commonly used to stain NMJ presynaptic terminals. Visual inspection of macro-annotated images still revealed errors in bouton counting for boutons that lacked a discernible interbouton space. All other NMJ features were displayed correctly, as is shown in a representative NMJ image (Panel A in S2 Fig). Neither the pre- or postsynaptic marker tested (Dlg1 and Hrp) where suitable for bouton counting with our macro. Since the number of boutons is a frequently assessed parameter in studies of NMJ morphology, we further optimized the macro in order to reliably recognize and count the boutons using the synaptic markers Synaptotagmin (Syt) and Cysteine string protein (Csp), two presynaptic vesicle-associated proteins. Both proved to be very suitable markers to distinguish and count even closely positioned boutons, probably because of the complete lack of staining in interbouton regions (Panels B-C in S2 Fig). For appropriate segmentation of bouton numbers a second macro was created: Drosophila_NMJ_Bouton_Morphometrics. It is available via the same public figshare repository: https://figshare.com/s/ec634918c027f62f7f2a [36].

Drosophila_NMJ_Bouton_Morphometrics allows users to accurately count boutons on Syt or Csp immunostaining, co-labeled with Brp The working procedure is the same as described previously for Drosophila_NMJ_Morphometrics (S1 Protocol). It provides a result file where the NMJ features: number of boutons, NMJ bouton area, NMJ length, NMJ longest branch length, number of islands, number of branches, number of branching points and number of active zones are assessed. To prove the reliability of Drosophila_NMJ_Bouton_Morphometrics bouton counts, the same validation procedure as described previously was used to evaluate manual versus macro bouton counts in n = 26 NMJ confocal images, labeled with Syt (S5 Table and S4 Fig). No significant differences in number of boutons and a ccc score of 0.96 between manual and macro counting were found (Table 3).

**Table 3:** Macro bouton validation using anti-synaptotagmin immunolabeled NMJs.

NMJ feature	Average manual count	Macro count	Significance (p-value)	Ccc	Average deviation	Sensitivity	Specificity
***Boutons***	27.88	27.15	0.76	0.96	x	96.21%	93.55%

## Discussion

Synapse morphology, shaped by synaptic transmission and regulating synaptic efficacy, is of central interest in neuroscience. However, it is still largely unknown how synapses adopt their overall shape. Most studies focused on one or few synaptic features rather than assessing synapse morphology more comprehensively and quantitatively. Here, we used the *Drosophila* larval NMJ, a widely used model for glutamatergic synapse biology and amenable to powerful genetic approaches, to pave the way for systematic synapse morphometry. We developed and released a Fiji-based macro to automatically and objectively evaluate nine morphological NMJ features. We estimate that the macro would save an experienced researcher up to 15 minutes per NMJ spent on manual image segmentation and analysis. By applying this method on a large number of muscle 4 glutamatergic type 1b NMJs, we quantified significant effects of gender, genetic background and abdominal body segment on multiple aspects of NMJ morphology. Correlation and PCA analyses demonstrated that the nine assessed morphological features can be grouped into five morphometric groups. The two groups that accounted for features involved in synaptic size and geometry contributed most to the first two principal components, which covered 60% of total variance. These two groups hardly correlated with each other. We propose that different molecular mechanisms control the two components, at least at the evaluated muscle 4 NMJ.

### Quantitative synapse morphometry

Our knowledge on molecules and mechanisms that shape synapses has greatly expanded in the last decade, and genetic screens in *Drosophila* have made an important contribution. Synapse morphology is a frequently used readout to discover genes required for proper synaptic function. Most studies have used (semi-)quantitative analyses, using e.g. the selection- and line-options of Fiji/ImageJ, but often only upon initial visual detection and performed by hand. Although this has proven to be sufficient to identify genes that if mutated grossly disrupt synapse development, this strategy likely has left more subtle modulators unidentified. Thus, the extent of the synapse regulome, including players that ensure proper orchestration of synapse coordinates, still awaits discovery. Their comprehensive identification needs a sensitive readout and a thorough understanding of how different morphological aspects relate to one another. Sutcliffe *et al*. created a publically available ImageJ plugin called “DeadEasy Synapse”, which measures the total voxel size of Brp-positive active zones per NMJ [44], and is thus complementary to our macro that instead achieves active zone counts and quantitative assessment of eight further morphometric NMJ features. In addition to Fiji/ImageJ, Cellprofiler is another open-source system for high-throughput image analysis [45]. It has been proven very useful for cell image analysis [46–48], but also for morphological phenotypes measured in *C*.*elegans* [49]. Cellprofiler lacks options to trace branch-like skeleton structures, required to measure NMJ length and branching pattern. In this study, we developed such a tool. We show that our Fiji-based macro is semi-automated, sensitive, and objective, whereas manual counting is laborious and can be assumed to be subject to interpersonal differences. The macro generates output files that allow the user to evaluate accuracy of the image segmentation and to correct or exclude (depending on the nature of the limiting feature) annotated images from further analysis. A low quality of the immunohistochemistry resulted in less correctly assessed NMJ images. Whenever the input quality was guaranteed (input checkpoints 1 and 2), we retained 84–92% of NMJ images, depending on the feature of interest. In this study where we took a specific interest in the correlations among all features, we exclusively used the NMJ images in which all features could be assessed with high accuracy. Staining variability is responsible for most of the excluded images. However, it similarly influences manual evaluation and can therefore not be linked to the macro performance.

Based on literature and our experimental observations, we aimed at quantifying nine morphological NMJ features. The macro performance was assessed for both wide field high-content and confocal muscle 4 NMJ images by investigating the deviation between manual and macro evaluations at three different levels: (1) sample distribution, (2) per given sample, and, most importantly, (3) for concordance. We deliberately chose the word ‘deviation’ over ‘error’ to underline that neither method can be considered as objectively true. Whereas successful for eight of these features, we were not able to optimize the bouton count in a satisfying manner for Dlg1, our marker of choice. The successful features were scored objectively and accurately, given the equal confidence interval widths and high concordance correlation coefficients (≥0.84, from which seven features even scored above 0.90). Whereas the macro measurements resulted in somewhat higher absolute values for length-related parameters, the high ccc scores demonstrate that this is a consistent proportional difference compared to the manual evaluation. Consequently, when both mutant and control samples are assessed by the same method, the difference (mutant:control) is equal for both methods. The macro counts somewhat higher absolute values, because it continuously thresholds between fore- and background, whereas manual measurements -in this case- are based on straight lines. The NMJ area is a two dimensional NMJ feature, a small difference in area evaluation therefore has a larger effect, explaining the somewhat lower ccc. This phenomenon is intrinsic to the nature of this parameter, as is also illustrated by a similar deviation between both manual experimentors. We conclude that our methodology shows accuracy and sensitivity comparable to manual evaluation for eight NMJ features.

Manual versus macro assessment of bouton number was not comparable, given the low concordance correlation coefficient of 0.22. We thus excluded the number of boutons from further analysis on this control dataset in this study. The macro uses a watershed transform -an algorithm that separates touching or slightly overlapping particles by identifying their local maxima in the distance function within these objects- on the segmented NMJ outline to distinguish individual boutons on NMJ outline invaginations, characteristic for interbouton regions [50]. We show that Dlg1 is not the optimal marker to determine bouton number, as this (mainly) postsynaptic marker presents with poorly pronounced interbouton constriction. We developed a second macro “Drosophila_NMJ_Bouton_Morphometrics” to assess the number of boutons using anti-Syt and anti-Csp, which successfully segmented boutons. We carefully validated the reliability of this second macro as done previously for our markers of interest (Dlg1 and Brp). Taken together, we developed 2 Fiji-based macros (Drosophila_NMJ_Morphometrics and Drosophila_NMJ_Bouton_Morphometrics) that perform objective and sensitive quantification of nine morphological NMJ features in a high-throughput manner.

### Sources of natural variation in synapse morphometry

Beyond the sensitive read-out that is required to detect subtle differences in synaptic morphology, a thorough understanding of natural variation and contributing factors is required to limit the calling of false positive phenotypes. We therefore quantified the effect of the main variables in our study -gender, genetic background and abdominal body segment- on the eight muscle 4 NMJ features acquired by our macro and the manually acquired muscle area.

Males and females showed considerable differences in size-related NMJ features and in the number of active zones. The female synaptic terminal was almost 5% longer and 8% bigger compared to males and it contained 7% more active zones. In agreement with the bigger size of female flies [51], the area of muscle 4 was 9% bigger in females than in males. Therefore, one might generate false positive results when comparing two datasets with each containing an uncontrolled amount of males-females. Interestingly, mutant screens or gene focused studies do not always report on gender selection or control, although gender can easily be selected for analyses [52]. Furthermore, gender selection is not mentioned in NMJ protocols that are often referred to by these studies [53–58]. Sex-specific differences at specific NMJ terminals were already described: Lnenicka, *et al* reported that muscle 5 produced larger excitatory postsynaptic potential in females and that type 1s motonerves on muscle 2 and 4 showed a greater charge transfer [59]. Interestingly, except for muscle size, no sex-specific difference in electrophysiology was detected for type 1b neurons on muscle 4, the synaptic terminal we focused on. Synapse morphology of 4-1s and 5-1b was measured, but no differences between males and females were found in this study for NMJ length, number of branches or number of boutons, which led the authors to suggest that the observed differences in transmitter release are due to ultra-structural and/or biochemical differences [59]. Our highly sensitive morphometry on 4-1b uncovered sex-specific NMJ properties that can potentially underlie the reported physiological differences. To our knowledge, no sex-specific regulators of NMJ morphology have been described yet.

Many of the NMJ-size related features were also significantly different between the two isogenic host strains (GD and KK RNAi libraries) we tested. Unquestionably, genetic differences can influence larval growth [60], but since no significant difference was observed in muscle 4 size, we did not find indication of overall differences in animal growth in the two investigated genotypes. A traceable difference between both strains is the *yellow+* marker in a *yellow* mutant background carried by the KK host strain. No NMJ abnormalities have been reported in *yellow* mutants, but *yellow* mutant alleles affect male courtship and mating behavior [61], suggesting a potential role in or effect on the nervous system.

Finally, we also demonstrated a significant impact of the abdominal body segments on muscle 4 NMJ morphometry. The observed patterns per morphological feature could be divided into four categories, with the greatest variance in geometry-related features and active zones. In general, muscle 4 type 1b synapses were shorter, more branched and have the highest number of active zones anteriorly.

In summary, all three tested variables in our NMJ analysis—gender, genetic background and abdominal segment- had a significant effect on at least some of the assessed morphological NMJ features on muscle 4. For quantitative evaluations, if aiming at high sensitivity, it is therefore important to take these features into account.

### NMJ size and geometry are differentially regulated

So far, *Drosophila* NMJ studies were often focused on a particular aspect of NMJ morphology, rather than assessing morphology more comprehensively. Consequently, the interdependencies of morphological features at this synapse, and at others, remained unknown. Carefully evaluating these relationships in unperturbed conditions, we here shed light onto which features are to what extent correlated, and thus provide first insights into the system properties of this important model synapse.

We identified five morphometric groups based on a pair wise correlation and principal component analysis. Interestingly, the features underlying the two groups explaining most of the variance hardly correlated, which led us to speculate that different biological processes underlie NMJ size and geometry. In agreement, both groups behaved differently between sexes and over the abdominal segments. Whereas NMJ geometry showed a decreasing number of branches and branching points from anterior to posterior, size features seemed to increase from abdominal segment 2 to 3.

Surprisingly, the muscle 4 area only correlated to a minor extent with NMJ size (R = 0.349) and very weakly with the number of active zone (R = 0.160). Although a strong correlation was observed between bouton number and muscle 6/7 size during embryonic and larval growth [62], the features seem to be less correlated within third instar larval stage. Whereas we cannot exclude differences between different NMJs/muscles, it seems more likely that the earlier observed correlation over a developmental time period reflect a general trend in growth to which all underlying mechanisms are subject to, rather than a tight causal relationship. Our result is in agreement with an earlier observation in which muscle size only partially (~50%) explained the variation in bouton number when comparing different *Drosophila* species [60]. Our finding provides an argument not to normalize synaptic size by muscle size, as has been practiced by some studies in third instar *Drosophila* larvae. These conclusions are applicable to NMJ morphological analyses of muscle 4 with the two markers of choice.

Although we validated our macro and carefully evaluated its performance in each NMJ image, our findings are still subject to technical variation such as variations in specimen dissections, immunolabeling intensities and the genome constitution. However, we have shown that the macro does not produce more variance than a manual counter does and the consistent correlations among gender and genetic background subgroups show the reproducibility of our data and support our conclusions. In our study, we evaluated three natural sources of variation to study the relation between nine synapse parameters: sex, abdominal segment identify and two selected genetic backgrounds. Additional sources causing NMJ structural variation have been demonstrated, such as larval and induced synapse activity [16,17]. We raised larvae under controlled conditions and therefore do not expect a significant variation in our data linked to such mechanisms.

**Fig 8:**
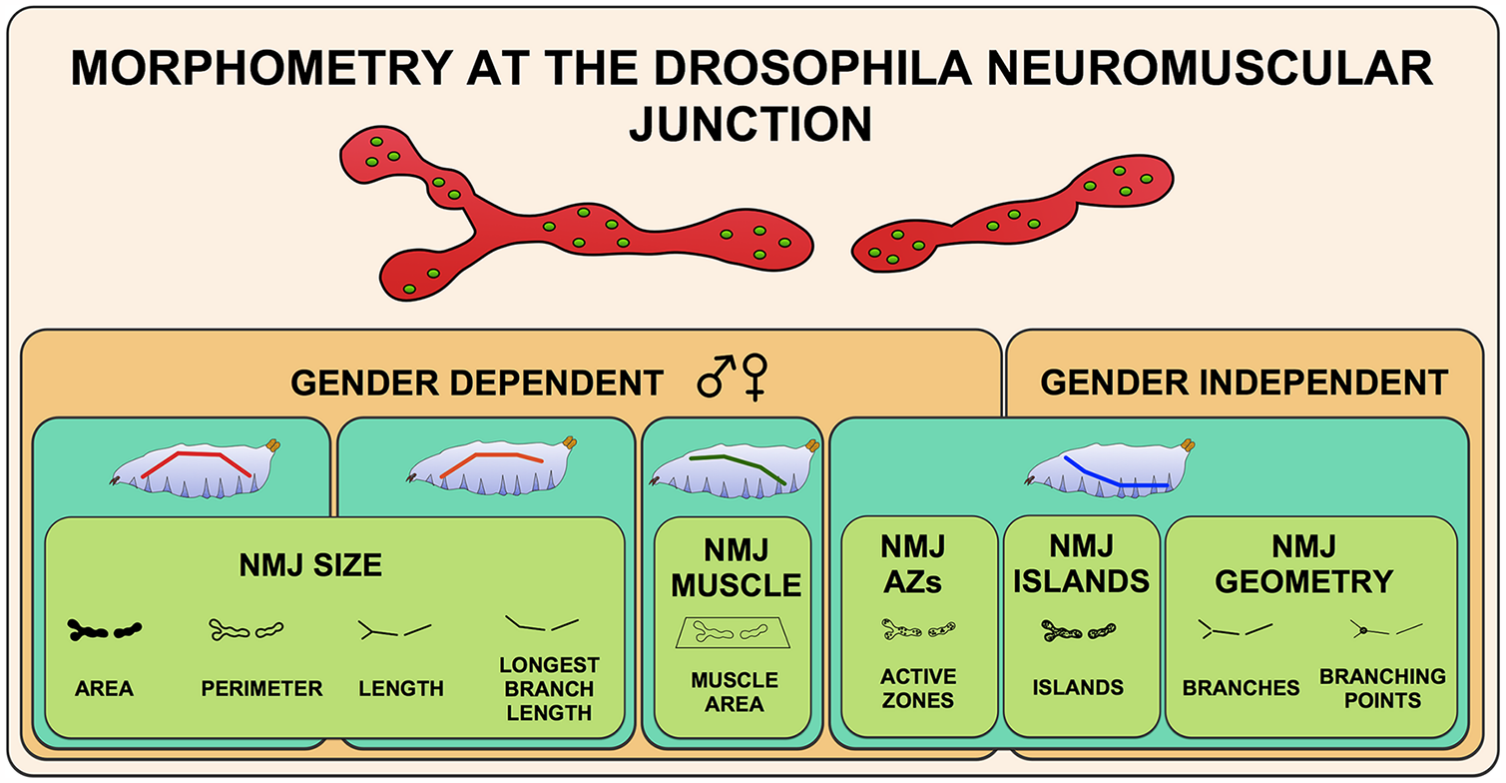
The different layers of *Drosophila* Neuromuscular Junction morphometry. The five principal components NMJ size, Muscle area, Active zones, Islands and NMJ geometry are indicated by the green boxes in the front layer. The second (turquoise) layer represents the different patterns in which the NMJ features behave over the four investigated body segments (A2-A5). The colored line in the larvae sketch shows the tendency of the parameters from anterior (A2, left) to posterior (A5, right). The third (orange) layer distinguishes the gender-dependent NMJ features from the gender independent ones. Variation by genetic background is not depicted since it depends on the identity of the specific genotypes investigated.

In summary, we developed a sensitive, accurate and semi-automated Fiji-based macro that permits high-throughput systems morphometry on the well-studied *Drosophila* larval NMJ on muscle 4. This method has the ability to handle several commonly used NMJ markers and microscope techniques. Here, we used a systems biology approach on two comprehensively generated, multiparametric datasets to start to understand how different morphological aspects of the synapse are coordinated. We showed how the nine measured morphometric features relate to one another and defined five morphometric groups in which features showed higher intra- than inter-correlation (Fig 8), suggesting that different molecular mechanisms are at work. The macro that we have developed and the here reported results have shed first light onto the design principles of an important model synapse, paved the way to quantitative synapse morphometry and can be applied to identify genes that couple features within a one morphometric group or that orchestrate the relations between different morphometric groups.

## Methods

### Fly stocks and maintenance

Fly stocks were maintained using standard *Drosophila* diet (sugar/cornmeal/yeast). Virgins from a *w*^*1118*^; UAS-*Dicer-2*; *elav-Gal4* promotor line were crossed to males from either *w*^*1118*^ (VDRC stock 60000 –genetic background of the GD library) or *y*,*w*^*1118*^*;P{attP*,*y[+]*,*w[3`]* (VDRC stock 60100 –genetic background of the KK library) and maintained at 28°C, 60% humidity. Crosses were performed with consistent amounts of flies and food. Positive control RNAi strains were in a similar manner crossed to virgins of the promotor line. The following RNAi strains were obtained at the Vienna *Drosophila* research center: Ankyrin2 (FBgn0261788; CG42734) *P[KK104937]VIE-260B* (VDRC stock KK107238) and *P[KK106729]VIE-260B* (VDRC stock KK107369); highwire (FBgn0030600; CG32592) *w*^*1118*^*; P[GD14101]v28163* (VDRC stock GD28163) and *w*^*1118*^*; P[GD14104]v36085* (VDRC stock GD36085) and Rab3 (FBgn0005586; CG7576) *P{KK108633}VIE-260B* (VDRC stock KK100787). Panneuronally induced knockdown conditions were compared to progenies from the driver crossed to the respective library host strain (GD60000 or KK60100).

### NMJ dissections & immunohistochemistry

Wandering L3 larvae were labeled for their gender and dissected, fixed in 3.7% paraformaldehyde for 30 min and co-labeled for bruchpilot (Brp) and discs large 1 (Dlg1). Brp was revealed using the primary antibody nc82 (1:125) (Developmental Studies Hybridoma Bank) applied overnight at 4°C and the secondary Alexa 488-labeled goat-anti-mouse antibody (1:125) (Invitrogen Molecular Probes). Discs large was visualized using primary antibody anti-Dlg1 (1:25) (Developmental Studies Hybridoma Bank) conjugated with the Zenon Alexa Fluor 568 Mouse IgG1 labeling kit (Invitrogen), applied according to the manufacturer’s protocol. For Hrp, Syt and Csp labeling, larvae were blocked for 1.5h on 5% NGS-PBS-T (0.3% Triton X-100 in PBS). Primary antibody anti-Hrp (rabbit, 1:750) (Jackson ImmunoResearch), anti-Syt (rabbit, 1:100) (kindly provided by H.Bellen) or anti-Csp (mouse, 1:25) was applied overnight at 4°C, followed by the Alexa 568- or 488-labeled secondary antibodies (1:500) (Invitrogen Molecular Probes).

### Image acquisition

NMJ images were obtained of type 1b NMJs at muscle 4 using an automated Leica DMI6000B high-content microscope. Individual NMJs were imaged at 10x (snapshot; Dlg1 only; 1.096 pixels/μm) and 63x magnification (stack; both channels; 6.932 pixels/μm). A fixed stack size was used, comprising 42 images per channel with a z-step size of 0.3μm and a z-volume of 12.152μm. The 2x42 images were saved as separate tiff files, encoding the NMJ number, z-plane and channel number in the file name. The area of muscle 4 was manually assessed via the segmented line option in Fiji at the lower magnification.

Confocal NMJ images were obtained of type 1b NMJs at muscle 4 using the Olympus FV1000 microscope. Individual NMJs were imaged at 60x (stack; both channels; 4.83 pixels/μm) with a z-step size of 0.91μm and a z-volume adjusted to the depth of the NMJ.

### Macro processing

The macro, written in ImageJ macro language, is compatible with the open source Fiji platform [32]. The entire analysis procedure consists of three steps, for which three separate sub-macros were written. This setup was chosen to allow maximum flexibility in the workflow. Sub macros can be executed from the main macro through a graphical user interface (GUI).

The first sub macro “Convert to stack” traverses a directory structure selected by the user in the GUI. Detected unprocessed images belonging to the same NMJ are recognized based on their z-plane and channel number and subsequently converted and saved to a hyperstack (containing all image data in a single tiff file) and maximum-intensity based Z-projection (referred to as ‘flat stack’). In our setup, the macro takes as input two channel z-stacks where the individual z-planes are stored as separate tiff files. The macro can however be adapted to deal with other types of input images.

The second part of the macro (sub macro 2 –“Define ROI”) is a semi-automated step where flat stacks are detected and opened automatically in a consecutive manner. Only images are opened that have not been processed by sub macro 2 before. In every flat stack, the region of interest (ROI) is manually defined by the user using the free hand selection tool. A binary ROI image is created and stored in the image source directory.

The third sub macro (“Analyze”) identifies hyperstacks for which the ROI image is present. NMJ image analysis is performed throughout each stack, within the limits of the image-specific ROI. Macro-annotated images are stored, containing a delineation of the analyzed NMJ. Additionally, a text file containing nine quantified features per NMJ is stored in the main directory.

### Macro image analysis

To enable high-throughput image acquisition and analysis, we used wide field fluorescence imaging to develop the macro. Out-of-focus fluorescence around the NMJ terminal, inherently present in these images, necessitates pre-processing to distinguish foreground signal from background noise. Therefore in the first step of the macro images are filtered applying a rolling ball background subtraction algorithm with a radius of 20 pixels. This algorithm is considered effective and fast for suppression of a non-uniform background with objects of rather constant diameter [63]. The outline of the entire NMJ is defined by an auto-threshold selection based on Renyi’s Entropy algorithm, applied to the Dlg1 or Hrp staining. This algorithm was shown to outperform several other entropy-based threshold selection methods [64], and resulted in consistent and adequate segmentation on a series of test images in the present study. Constriction of the synaptic terminal between boutons provided the basis for the analysis of bouton counting’s. A watershed separation is performed on the binary NMJ outline. Resulting objects exceeding an (empirically determined lower bound) area threshold of 100 pixels are considered to represent boutons. To filter against background noise as for example present in Hrp staining, an optional filter (“Remove small particles”) was implemented to remove particles smaller than 100 pixels.

To measure NMJ length and branching geometry, a binary skeleton for the NMJ is determined. The skeleton is a one-pixel thick axis along the center of the NMJ, calculated using mathematical morphology on the binary image. We found that the auto-threshold described above, used to accurately determine the NMJ outline, and was sometimes too restricted for accurate determination of the skeleton when using Dlg1, Syt or Csp staining. The macro therefore uses auto-threshold selection based on Li’s Minimum Cross Entropy for this purpose. This algorithm generally results in somewhat wider segmentation results, as previously witnessed by the results of Sengur et al. [65]. The Renyi’s Entropy algorithm was used for NMJs stained with Hrp. From the NMJ skeleton five features (length, longest branch length, number of branches, branching points and islands) are calculated.

Subsequently, the number of active zones is counted in the Brp-channel by finding local intensity maxima in the 3D image stack. To reduce the effect of intensity variations over individual active zones, stacks are first filtered applying a 3D grey closing with a small circular structuring element. Identified local maxima are considered to represent one active zone if they do not touch other local maxima (either horizontally/vertically or diagonally) and exceed a minimum intensity level (automatically determined using Huang's fuzzy thresholding method), to prevent background fluctuations to be counted as active zones.

Confocal NMJ images were processed in a similar manner, with maxima noise tolerance ‘100’ and Brp-puncta lower threshold ‘250’.

### Macro validation

The nine NMJ features measured by the macro were in parallel manually quantified, blind to the macro results, independently by two experimenters in 30 NMJ images. Images were processed with Fiji. For each channel a projection was created from in-focus planes. The subtract background algorithm was applied to the Dlg1 channel, followed by 3 consecutive applications of the standard FIJI smooth filter (3x3 average filter), and area and perimeter were determined by manually thresholding the NMJ terminals. All length related NMJ features were measured using the freehand lines tool. Active zones were visually assessed in the Brp channel. Macro counts were plotted against averaged manual counts, and Lin’s concordance correlation coefficients was calculated in R, using the epi.ccc function of the epiR package [66]. The % deviation between manual and macro count is calculated as (average manual result—macro result) / average manual result x 100%. Sensitivity is true positives / (true positives + false positives), specificity is true positives / (true positives + false negatives). Active zone results are compared to experimentor #1.

Confocal NMJ images (n = 15) were validated in a similar manner by one experimenter. Picture one was excluded from Brp-analysis because of low staining quality.

### Statistics

Statistics were performed in R (R Development Core Team, 2008) [67]. For comparisons between manual and macro counts, gender and genetic backgrounds, independent 2-group t-tests were applied for normally distributed features (area, active zones, boutons, length, longest branch length, perimeter and muscle) and Mann-Whitney U tested for not normally distributed features (branches, branching points and islands). Whenever required, p-values were adjusted by a Holm-Bonferroni correction and indicated as padj. Anova-Tukey method was used for body segment analysis followed by Tukey’s honest significance test (Tukey’s HSD). Pearson correlations were calculated for the different feature combinations and visualized in an adjusted scatter plot matrix [68]. Principal component analysis [69] was used to study the relationship among different aspects of synapse morphology.

### Drosophila_NMJ_Bouton_Morphometrics

Drosophila_NMJ_Bouton_Morphometrics was specifically developed to assess the number of NMJ boutons of Syt- or CSP- immunostained NMJs. The macro processing and macro analysis follow the same steps as described previously in this section, except that the outline of the entire NMJ is defined by an auto-threshold selection based on the algorithm moments and a dilating step prior to the watershed separation. These increase efficiency of bouton segmentation. The algorithm area in this macro assesses bouton area after the bouton segmentation, and the algorith NMJ perimeter is obsolete and has been removed. Resulting objects exceeding an (empirically determined lower bound) area threshold of 10 pixels are considered to represent boutons. The filter against background noise (“Remove small particles”) should always be activated when running this macro and was implemented to remove particles smaller than 10 pixels.

The validation of bouton counts for the Drosophila_NMJ_Bouton_Morphometrics macro was performed using confocal images of NMJs (n = 26) immunolabeled with anti-Syt antibody. The number of boutons were counted by two experimenters blind to the results of the macro, and where compared with the macro counts using the same procedures as described in macro validation. Two picture where excluded from Syt-analysis because of low staining quality.

## Supporting Information

S1 Table Macro validation summary (results per NMJ image, high-content microscopy). (XLSX)

S2 Table Statistical summary NMJ features (groups and abdominal segments). (XLSX)

S3 Table Principal component analysis summary (groups and abdominal segments). (XLSX)

S4 Table Macro validation summary (results per NMJ image). (XLSX)

S5 Table Macro validation summary (results per NMJ image, bouton counting using Drosophila_NMJ_Bouton_Morphometrics macro on Syt immunolabeled NMJs). (XLSX)

S1 Fig Macro versus manual evaluation. The plots display the mean value (black dot) and 95% confidence interval for each NMJ feature. Manual experimentors are color-coded by blue and red (experimentor #1 and #2, respectively) and the macro by green.
(EPS)

S2 Fig Applicability of the macro to Hrp, Syt and Csp markers. Representative muscle 4 NMJ images of the marker (left side) and the macro-annotated image (right side) for the markers Hrp (A-A’), Syt (B-B’) and Csp (C-C’).
(EPS)

S3 Fig Applicability of the macro on confocal NMJ images. Representative muscle 4 confocal NMJ images of the marker (left side) and the macro-annotated image (right side) for the markers Dlg1 (A-A’), Brp (B-B’) and Hrp (C-C’).
(EPS)

S4 Fig Drosophila_NMJ_Bouton_Morphometrics bouton counts versus manual evaluation. The graph represents the concordance correlation coefficient (ccc) between manually assessed ((x-axis) and macro-based bouton counts (y-axis). (A) Each data point represents the macro and manual measurement for a given NMJ(n = 26). (B) displayed are the mean value (black dot) and 95% confidence interval for bouton counts by two manual experimentors (blue and red, experimentor #1 and #2, respectively) and by the macro in green.
(TIF)

S1 Protocol Step-by-step guide for multiparametric analyses of NMJs with Drosophila_NMJ_morphometrics and Drosophila_NMJ_bouton_morphometrics macros. (DOCX)

## References

[1] Lin YC, Koleske AJ (2010) Mechanisms of synapse and dendrite maintenance and their disruption in psychiatric and neurodegenerative disorders. Annu Rev Neurosci 33: 349–378. 10.1146/annurev-neuro-060909-153204 20367247 PMC3063389

[2] van Bokhoven H (2011) Genetic and epigenetic networks in intellectual disabilities. Annu Rev Genet 45: 81–104. 10.1146/annurev-genet-110410-132512 21910631

[3] Penzes P, Buonanno A, Passafaro M, Sala C, Sweet RA (2013) Developmental vulnerability of synapses and circuits associated with neuropsychiatric disorders. J Neurochem 126: 165–182. 10.1111/jnc.12261 23574039 PMC3700683

[4] Wondolowski J, Dickman D (2013) Emerging links between homeostatic synaptic plasticity and neurological disease. Front Cell Neurosci 7: 223 10.3389/fncel.2013.00223 24312013 PMC3836049

[5] Banerjee S, Riordan M, Bhat MA (2014) Genetic aspects of autism spectrum disorders: insights from animal models. Front Cell Neurosci 8: 58 10.3389/fncel.2014.00058 24605088 PMC3932417

[6] Mainen ZF, Sejnowski TJ (1996) Influence of dendritic structure on firing pattern in model neocortical neurons. Nature 382: 363–366. 8684467 10.1038/382363a0

[7] Yuste R, Majewska A, Holthoff K (2000) From form to function: calcium compartmentalization in dendritic spines. Nat Neurosci 3: 653–659. 10862697 10.1038/76609

[8] Vetter P, Roth A, Hausser M (2001) Propagation of action potentials in dendrites depends on dendritic morphology. J Neurophysiol 85: 926–937. 11160523 10.1152/jn.2001.85.2.926

[9] Dahlhaus M, Levelt CN (2010) Structure and function relationships during ocular dominance plasticity in the visual cortex. Rev Neurosci 21: 223–237. 20879693 10.1515/revneuro.2010.21.3.223

[10] Bosch M, Hayashi Y (2012) Structural plasticity of dendritic spines. Curr Opin Neurobiol 22: 383–388. 10.1016/j.conb.2011.09.002 21963169 PMC4281347

[11] Fortin DA, Srivastava T, Soderling TR (2012) Structural modulation of dendritic spines during synaptic plasticity. Neuroscientist 18: 326–341. 10.1177/1073858411407206 21670426

[12] Mehnert KI, Cantera R (2011) Circadian rhythms in the morphology of neurons in Drosophila. Cell Tissue Res 344: 381–389. 10.1007/s00441-011-1174-x 21562943

[13] Gorska-Andrzejak J, Makuch R, Stefan J, Gorlich A, Semik D, et al (2013) Circadian expression of the presynaptic active zone protein Bruchpilot in the lamina of Drosophila melanogaster. Dev Neurobiol 73: 14–26. 10.1002/dneu.22032 22589214

[14] Muraro NI, Pirez N, Ceriani MF (2013) The circadian system: plasticity at many levels. Neuroscience 247: 280–293. 10.1016/j.neuroscience.2013.05.036 23727010

[15] Ruiz S, Ferreiro MJ, Menhert KI, Casanova G, Olivera A, et al (2013) Rhythmic changes in synapse numbers in Drosophila melanogaster motor terminals. PLoS One 8: e67161 10.1371/journal.pone.0067161 23840613 PMC3695982

[16] Sigrist SJ, Reiff DF, Thiel PR, Steinert JR, Schuster CM (2003) Experience-dependent strengthening of Drosophila neuromuscular junctions. J Neurosci 23: 6546–6556. 12878696 10.1523/JNEUROSCI.23-16-06546.2003 PMC6740647

[17] Ataman B, Ashley J, Gorczyca M, Ramachandran P, Fouquet W, et al (2008) Rapid activity-dependent modifications in synaptic structure and function require bidirectional Wnt signaling. Neuron 57: 705–718. 10.1016/j.neuron.2008.01.026 18341991 PMC2435264

[18] Ruiz-Canada C, Budnik V (2006) Introduction on the use of the Drosophila embryonic/larval neuromuscular junction as a model system to study synapse development and function, and a brief summary of pathfinding and target recognition. Int Rev Neurobiol 75: 1–31. 17137921 10.1016/S0074-7742(06)75001-2

[19] Menon KP, Carrillo RA, Zinn K (2013) Development and plasticity of the Drosophila larval neuromuscular junction. Wiley Interdiscip Rev Dev Biol 2: 647–670. 10.1002/wdev.108 24014452 PMC3767937

[20] Collins CA, DiAntonio A (2007) Synaptic development: insights from Drosophila. Curr Opin Neurobiol 17: 35–42. 17229568 10.1016/j.conb.2007.01.001

[21] Atwood HL, Govind CK, Wu CF (1993) Differential ultrastructure of synaptic terminals on ventral longitudinal abdominal muscles in Drosophila larvae. J Neurobiol 24: 1008–1024. 8409966 10.1002/neu.480240803

[22] Kraut R, Menon K, Zinn K (2001) A gain-of-function screen for genes controlling motor axon guidance and synaptogenesis in Drosophila. Curr Biol 11: 417–430. 11301252 10.1016/s0960-9822(01)00124-5

[23] Parnas D, Haghighi AP, Fetter RD, Kim SW, Goodman CS (2001) Regulation of postsynaptic structure and protein localization by the Rho-type guanine nucleotide exchange factor dPix. Neuron 32: 415–424. 11709153 10.1016/s0896-6273(01)00485-8

[24] Aberle H, Haghighi AP, Fetter RD, McCabe BD, Magalhaes TR, et al (2002) wishful thinking encodes a BMP type II receptor that regulates synaptic growth in Drosophila. Neuron 33: 545–558. 11856529 10.1016/s0896-6273(02)00589-5

[25] Eaton BA, Fetter RD, Davis GW (2002) Dynactin is necessary for synapse stabilization. Neuron 34: 729–741. 12062020 10.1016/s0896-6273(02)00721-3

[26] Rohrbough J, Rushton E, Palanker L, Woodruff E, Matthies HJ, et al (2004) Ceramidase regulates synaptic vesicle exocytosis and trafficking. J Neurosci 24: 7789–7803. 15356190 10.1523/JNEUROSCI.1146-04.2004 PMC2675194

[27] Laviolette MJ, Nunes P, Peyre JB, Aigaki T, Stewart BA (2005) A genetic screen for suppressors of Drosophila NSF2 neuromuscular junction overgrowth. Genetics 170: 779–792. 15834148 10.1534/genetics.104.035691 PMC1450403

[28] Collins CA, Wairkar YP, Johnson SL, DiAntonio A (2006) Highwire restrains synaptic growth by attenuating a MAP kinase signal. Neuron 51: 57–69. 16815332 10.1016/j.neuron.2006.05.026

[29] Liebl FL, Werner KM, Sheng Q, Karr JE, McCabe BD, et al (2006) Genome-wide P-element screen for Drosophila synaptogenesis mutants. J Neurobiol 66: 332–347. 16408305 10.1002/neu.20229 PMC1626350

[30] Valakh V, Naylor SA, Berns DS, DiAntonio A (2012) A large-scale RNAi screen identifies functional classes of genes shaping synaptic development and maintenance. Dev Biol 366: 163–171. 10.1016/j.ydbio.2012.04.008 22542760 PMC3358632

[31] Blunk AD, Akbergenova Y, Cho RW, Lee J, Walldorf U, et al (2014) Postsynaptic actin regulates active zone spacing and glutamate receptor apposition at the Drosophila neuromuscular junction. Mol Cell Neurosci 61: 241–254. 10.1016/j.mcn.2014.07.005 25066865 PMC4134997

[32] Schindelin J, Arganda-Carreras I, Frise E, Kaynig V, Longair M, et al (2012) Fiji: an open-source platform for biological-image analysis. Nat Methods 9: 676–682. 10.1038/nmeth.2019 22743772 PMC3855844

[33] Dietzl G, Chen D, Schnorrer F, Su KC, Barinova Y, et al (2007) A genome-wide transgenic RNAi library for conditional gene inactivation in Drosophila. Nature 448: 151–156. 17625558 10.1038/nature05954

[34] Wagh DA, Rasse TM, Asan E, Hofbauer A, Schwenkert I, et al (2006) Bruchpilot, a protein with homology to ELKS/CAST, is required for structural integrity and function of synaptic active zones in Drosophila. Neuron 49: 833–844. 16543132 10.1016/j.neuron.2006.02.008

[35] Kittel RJ, Wichmann C, Rasse TM, Fouquet W, Schmidt M, et al (2006) Bruchpilot promotes active zone assembly, Ca2+ channel clustering, and vesicle release. Science 312: 1051–1054. 16614170 10.1126/science.1126308

[36] Nijhof B, Castells-Nobau A, Wolf L, Scheffer-de Gooyert JM, Monedero I, et al (2016) Drosophila NMJ Morphometrics. 10.1371/journal.pcbi.1004823 PMC4801422 26998933

[37] Lin LI (1989) A concordance correlation coefficient to evaluate reproducibility. Biometrics 45: 255–268. 2720055

[38] Koch I, Schwarz H, Beuchle D, Goellner B, Langegger M, et al (2008) Drosophila ankyrin 2 is required for synaptic stability. Neuron 58: 210–222. 10.1016/j.neuron.2008.03.019 18439406

[39] Pielage J, Cheng L, Fetter RD, Carlton PM, Sedat JW, et al (2008) A presynaptic giant ankyrin stabilizes the NMJ through regulation of presynaptic microtubules and transsynaptic cell adhesion. Neuron 58: 195–209. 10.1016/j.neuron.2008.02.017 18439405 PMC2699047

[40] Iqbal Z, Vandeweyer G, van der Voet M, Waryah AM, Zahoor MY, et al (2013) Homozygous and heterozygous disruptions of ANK3: at the crossroads of neurodevelopmental and psychiatric disorders. Hum Mol Genet 22: 1960–1970. 10.1093/hmg/ddt043 23390136

[41] Wan HI, DiAntonio A, Fetter RD, Bergstrom K, Strauss R, et al (2000) Highwire regulates synaptic growth in Drosophila. Neuron 26: 313–329. 10839352 10.1016/s0896-6273(00)81166-6

[42] Graf ER, Daniels RW, Burgess RW, Schwarz TL, DiAntonio A (2009) Rab3 dynamically controls protein composition at active zones. Neuron 64: 663–677. 10.1016/j.neuron.2009.11.002 20005823 PMC2796257

[43] Prokop A (2006) Organization of the efferent system and structure of neuromuscular junctions in Drosophila. Int Rev Neurobiol 75: 71–90. 17137924 10.1016/S0074-7742(06)75004-8

[44] Sutcliffe B, Forero MG, Zhu B, Robinson IM, Hidalgo A (2013) Neuron-type specific functions of DNT1, DNT2 and Spz at the Drosophila neuromuscular junction. PLoS One 8: e75902 10.1371/journal.pone.0075902 24124519 PMC3790821

[45] Carpenter AE, Jones TR, Lamprecht MR, Clarke C, Kang IH, et al (2006) CellProfiler: image analysis software for identifying and quantifying cell phenotypes. Genome Biol 7: R100 17076895 10.1186/gb-2006-7-10-r100 PMC1794559

[46] Mittag A, Pinto FE, Endringer DC, Tarnok A, Lenz D (2011) Cellular analysis by open-source software for affordable cytometry. Scanning 33: 33–40. 10.1002/sca.20220 21319173

[47] Osaka I, Hills JM, Kieweg SL, Shinogle HE, Moore DS, et al (2012) An automated image-based method for rapid analysis of Chlamydia infection as a tool for screening antichlamydial agents. Antimicrob Agents Chemother 56: 4184–4188. 10.1128/AAC.00427-12 22615279 PMC3421616

[48] Gonzalez JE, Lee M, Barquinero JF, Valente M, Roch-Lefevre S, et al (2012) Quantitative image analysis of gamma-H2AX foci induced by ionizing radiation applying open source programs. Anal Quant Cytol Histol 34: 66–71. 22611761

[49] Wahlby C, Kamentsky L, Liu ZH, Riklin-Raviv T, Conery AL, et al (2012) An image analysis toolbox for high-throughput C. elegans assays. Nat Methods 9: 714–716. 10.1038/nmeth.1984 22522656 PMC3433711

[50] Vincent L, Soille P (1991) Watersheds in digital spaces: an efficient algorithm based on immersion simulations. Pattern Analysis and Machine Intelligence, IEEE Transactions 13: 583–598.

[51] Mirth CK, Shingleton AW (2012) Integrating body and organ size in Drosophila: recent advances and outstanding problems. Front Endocrinol (Lausanne) 3: 49. 22654869 10.3389/fendo.2012.00049 PMC3356080

[52] Demerec M, Kaufmann BP (1996) Drosophila Guide: Introduction to the Genetics and Cytology of *Drosophila melanogaster*. Tenth Edition Washington, D.C: Carnegie Institution of Washington.

[53] Bellen HJ, Budnik V (2000) *Drosophila* protocols, chapter 11: The Neuromuscular Junction. New York: Cold Spring Harbor Laboratory Press.

[54] Budnik V, Gorczyca M, Prokop A (2006) Selected methods for the anatomical study of Drosophila embryonic and larval neuromuscular junctions. Int Rev Neurobiol 75: 323–365. 17137935 10.1016/S0074-7742(06)75015-2

[55] Brent JR, Werner KM, McCabe BD (2009) Drosophila larval NMJ dissection. J Vis Exp. 10.3791/1107 PMC2762896 19229190

[56] Brent J, Werner K, McCabe BD (2009) Drosophila larval NMJ immunohistochemistry. J Vis Exp. 10.3791/1108 PMC2762900 19329927

[57] Ramachandran P, Budnik V (2010) *Drosophila* Neurobiology A laboratory manual, chapter 7: Enmbryonic and Larval Neuromuscular Junction: An overview with Selected Methods and Protocols. New York: Cold Spring Harbor Laboratory Press.

[58] Smith R, Taylor JP (2011) Dissection and imaging of active zones in the Drosophila neuromuscular junction. J Vis Exp. 10.3791/2676 PMC3169289 21559003

[59] Lnenicka GA, Theriault K, Monroe R (2006) Sexual differentiation of identified motor terminals in Drosophila larvae. J Neurobiol 66: 488–498. 16470738 10.1002/neu.20234

[60] Campbell M, Ganetzky B (2012) Extensive morphological divergence and rapid evolution of the larval neuromuscular junction in Drosophila. Proc Natl Acad Sci U S A 109: E648–655. 10.1073/pnas.1201176109 22355119 PMC3306701

[61] St Pierre SE, Ponting L, Stefancsik R, McQuilton P, FlyBase C (2014) FlyBase 102—advanced approaches to interrogating FlyBase. Nucleic Acids Res 42: D780–788. 10.1093/nar/gkt1092 24234449 PMC3964969

[62] Schuster CM, Davis GW, Fetter RD, Goodman CS (1996) Genetic dissection of structural and functional components of synaptic plasticity. I. Fasciclin II controls synaptic stabilization and growth. Neuron 17: 641–654. 8893022 10.1016/s0896-6273(00)80197-x

[63] Sternberg SR (1983) Biomedical Image Processing. Computer 16: 22–34.

[64] Sahoo P, Wilkins C, Yeager J (1997) Threshold selection using Renyi's entropy. Pattern Recognition 30: 71–84.

[65] Şengür A, Türkoğlu İ, ve İnce MC (2006) A Comparative Study On Entropic Thresholding Methods. Istanbul University—Journal of Electrical & Electronics Engineering 6: 183–188.

[66] Stevenson M, Nunes T, Heuer C, Marshall J, Sanchez J, et al. (2014) epiR: An R package for the analysis of epidemiological data. R package version 0.9–59. The Comprehensive R Archive Network.

[67] R-Development-Core-Team (2014) R: a language and environment for statistical computing R Foundation for Statistical Computing, Vienna, Austria.

[68] Hurley C (2012) gclus: Clustering Graphics. R package version 1.3.1.

[69] Husson FJ, J.; Le, S.; Mazet, J. (2014) FactoMineR: Multivariate Exploratory Data Analysis and Data Mining with R. R package version 1.26.

