# Supplementary figures and images for: A New Fiji-Based Algorithm That Systematically Quantifies Nine Synaptic Parameters Provides Insights into Drosophila NMJ Morphometry
Source: PLoS Comput Biol. 2016 Mar 21;12(3):e1004823. doi: 10.1371/journal.pcbi.1004823 (PMC4801422; doi:10.1371/journal.pcbi.1004823)

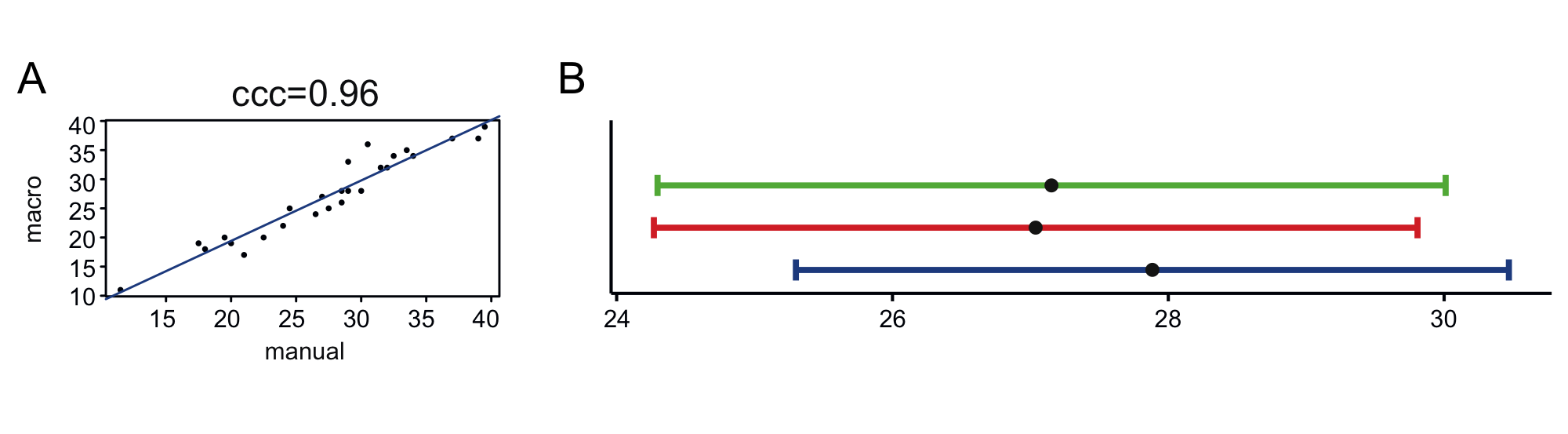

Supplement: S4 Fig — The graph represents the concordance correlation coefficient (ccc) between manually assessed ((x-axis) and macro-based bouton counts (y-axis). (A) Each data point represents the macro and manual measurement for a given NMJ(n = 26). (B) displayed are the mean value (black dot) and 95% confidence interval for bouton counts by two manual experimentors (blue and red, experimentor #1 and #2, respectively) and by the macro in green. (TIF) [file pcbi.1004823.s009.tif]
