# Supplementary material for: A New Fiji-Based Algorithm That Systematically Quantifies Nine Synaptic Parameters Provides Insights into Drosophila NMJ Morphometry
Source: PLoS Comput Biol. 2016 Mar 21;12(3):e1004823. doi: 10.1371/journal.pcbi.1004823 (PMC4801422; doi:10.1371/journal.pcbi.1004823)

**Protocol S1. Fiji macros ‘Drosophila_NMJ_Morphometrics’ and ‘Drosophila_NMJ_Bouton Morphometrics’**

*Nijhof et al. A new Fiji-based algorithm that systematically quantifies nine synaptic parameters provides insights into Drosophila NMJ morphometry. 2016*

Content:

‘Drosophila_NMJ_Morphometrics’ or ‘Drosophila_NMJ_Bouton_Morphometrics’ -

which macro to apply 2

Install Fiji and download the macro into the Fiji plugins folder 3

Prepare your NMJ images 4

Start the graphical interface 5

Sub macros 5

Choose settings in the graphical interface 6

Run sub macro ‘Convert to stack’ 8

How to deal with images that are not stores as a ‘.tif separated files 9

Change channel order 10

Run sub macro ‘Define ROI’ 11

Run sub macro ‘Analysis’ 13

Settings used for this paper 16

**
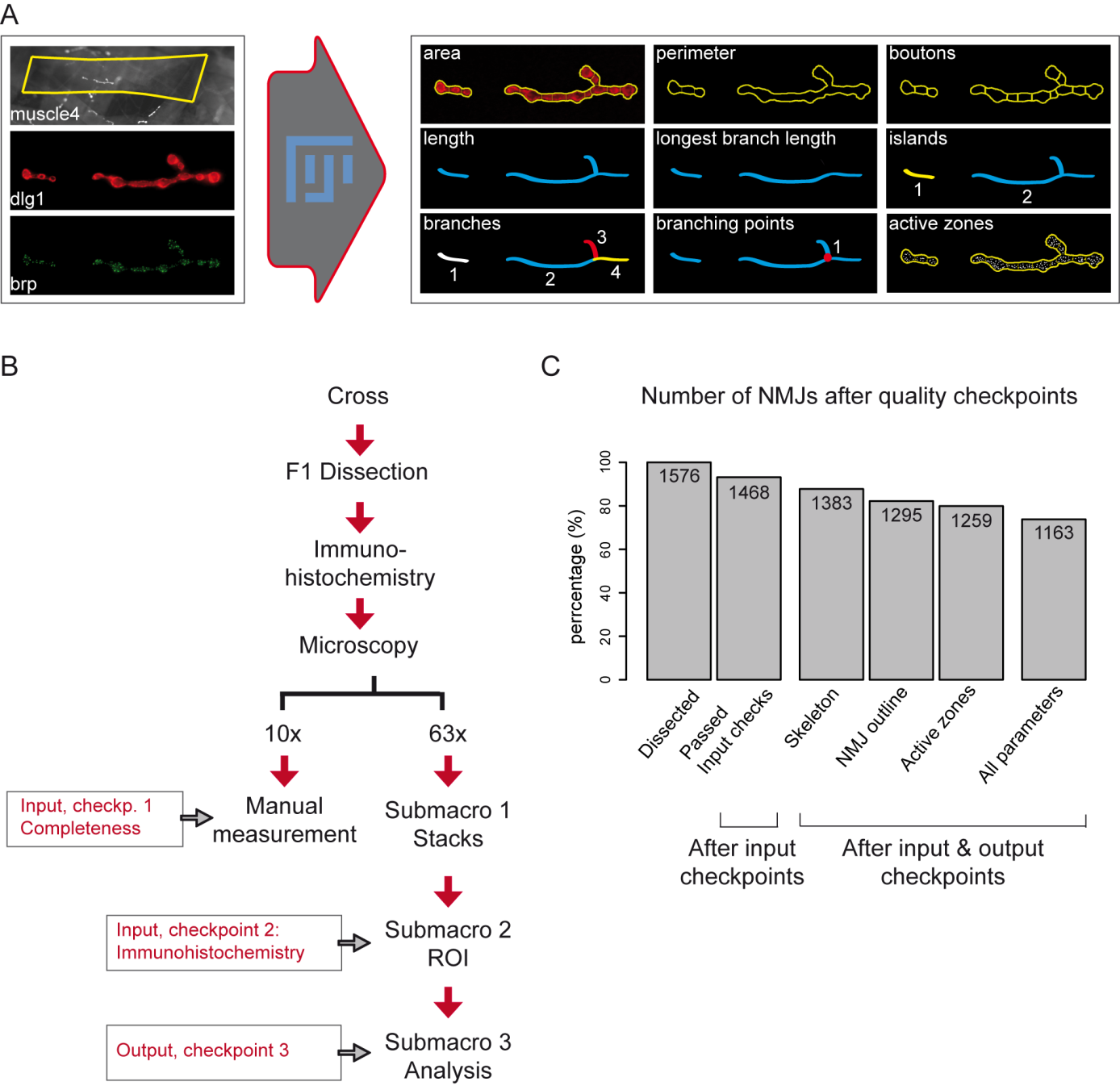
**

**■ ‘Drosophila_NMJ_Morphometrics’ or ‘Drosophila_NMJ_Bouton_Morphometrics’ - which macro to apply**

**Drosophila_NMJ_Morphometrics** measures NMJ terminals immunostained with Dlg1- or Hrp (channel 1), and Brp (channel 2) labelings. It quantifies the following parameters: NMJ area, NMJ perimeter, number of boutons, NMJ length, NMJ longest branch length, number of islands, number of branches, number of branching points and number of active zones in the NMJ terminal determined by the user. Drosophila_NMJ_Morphometrics has been validated to accurately measure all underlined parameters on Dlg1 and Hrp immunolabeling as described in our research paper. Although the algorithm to determine the number of boutons has been retained in this macro, it unfortunately generates non-satisfying numbers of bouton counts.

To properly assess the number of boutons it is necessary to use the **Drosophila_NMJ_Bouton_Morphometrics** macro, which was specifically generated to correctly quantify boutons, using Syt- or Csp- (channel 1) and Brp (channel 2) immunostained NMJ images. **Drosophila_NMJ_Bouton_Morphometrics** quantifies the following parameters: number of boutons, NMJ bouton area, NMJ length, NMJ longest branch length, number of islands, number of branches, number of branching points and number of active zones in the NMJ terminal determined by the user. The bouton counts by **Drosophila_NMJ_Bouton_Morphometrics** have been validated for NMJ terminals immunolabeled with Syt, as described in our research paper. The other parameters (NMJ length, NMJ longest branch length, number of islands, number of branches, number of branching points) have not been validated on Syt immunostaining, however, the algorithms applied to determine these parameters in **Drosophila_NMJ_Bouton_Morphometrics** do not differ from the ones applied in **Drosophila_NMJ_Morphometrics.**

In summary, validated, accurate measurements of NMJ area, NMJ perimeter, NMJ length, NMJ longest branch length, number of islands, number of branches, number of branching points and number of active zones can be obtained using the **Drosophila_NMJ_Morphometrics** macro on Dlg1 or Hrp (channel 1), and Brp (channel 2) labeled NMJs, whereas validated, accurate measurements of number of boutons and bouton area can be obtained using the **Drosophila_NMJ_Bouton_Morphometrics** macro on Syt- or Csp- (channel 1) and Brp (channel 2) immunostained NMJs.

**
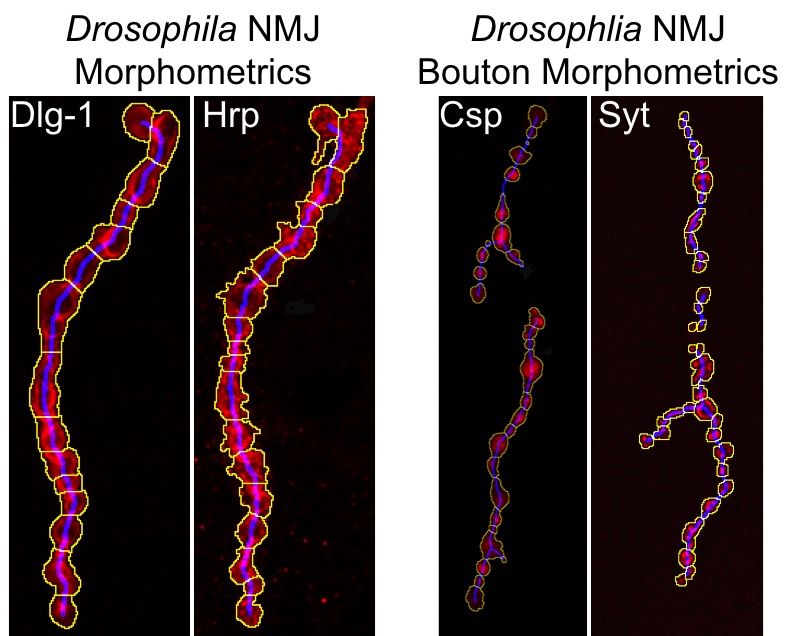
**

**■ Install Fiji and download the macro into the Fiji plugins folder**

1. To download Fiji, follow the following link and its download instructions:

‘Drosophila_NMJ_Morphometrics’ and ‘Drosophila_NMJ_Bouton_Morphometrics’ are compatible with Fiji versions 1.3 and 1.4. Compatible Fiji versions can be found under the name **Fiji Life-Line version, 2014 November 25** at the following link: ‘<http://fiji.sc/Downloads#Life-Line_Fiji_versions>’. A copy of the required Fiji version is also available in our repository (see below).

To check which Fiji version is installed on your computer:

Fiji menu: Help>>About ImageJ… A window will appear indicating the Fiji and Java version installed.

Note: It is of crucial importance to adhere to the indicated Fiji versions. Newer or older Fiji versions may lead to errors (such as "**image not found in line 304**" upon use of Fiji 1.5 on PC) when running the macro(s). Automatic update inquiries of Fiji therefore always need to be rejected.

2. The macros ‘Drosophila_NMJ_Morphometrics’ and ‘Drosophila_NMJ_Bouton_Morphometrics’ are available via figshare, a public repository where users can make their research outputs available. The macro is citable by its unique DOI:10.6084/m9.figshare.2077399 (‘Digital Object Identifier’). The corresponding DOI link is:

[**https://figshare.com/s/ec634918c027f62f7f2a**](https://figshare.com/s/ec634918c027f62f7f2a)

Note: This repository contains the macros **‘Drosophila_NMJ_Morphometrics’** and **‘Drosophila_NMJ_Bouton_Morphometrics’**, a copy of a compatible Fiji version for PC and Mac and three example folders (Example 1-3), which each contain 2 NMJ image series. These files, together with the accompanying document ‘Examples Guide’ allows the user to quickly familiarize with the image import options and potentially required image adjustments to run the macros.

3. ‘Drosophila_NMJ_Morphometrics’ and ‘Drosophila_NMJ_Bouton_Morphometrics’ are compatible with PC and MAC.

4. Download the macro into the plugins folder of Fiji (called ‘Fiji.app’).


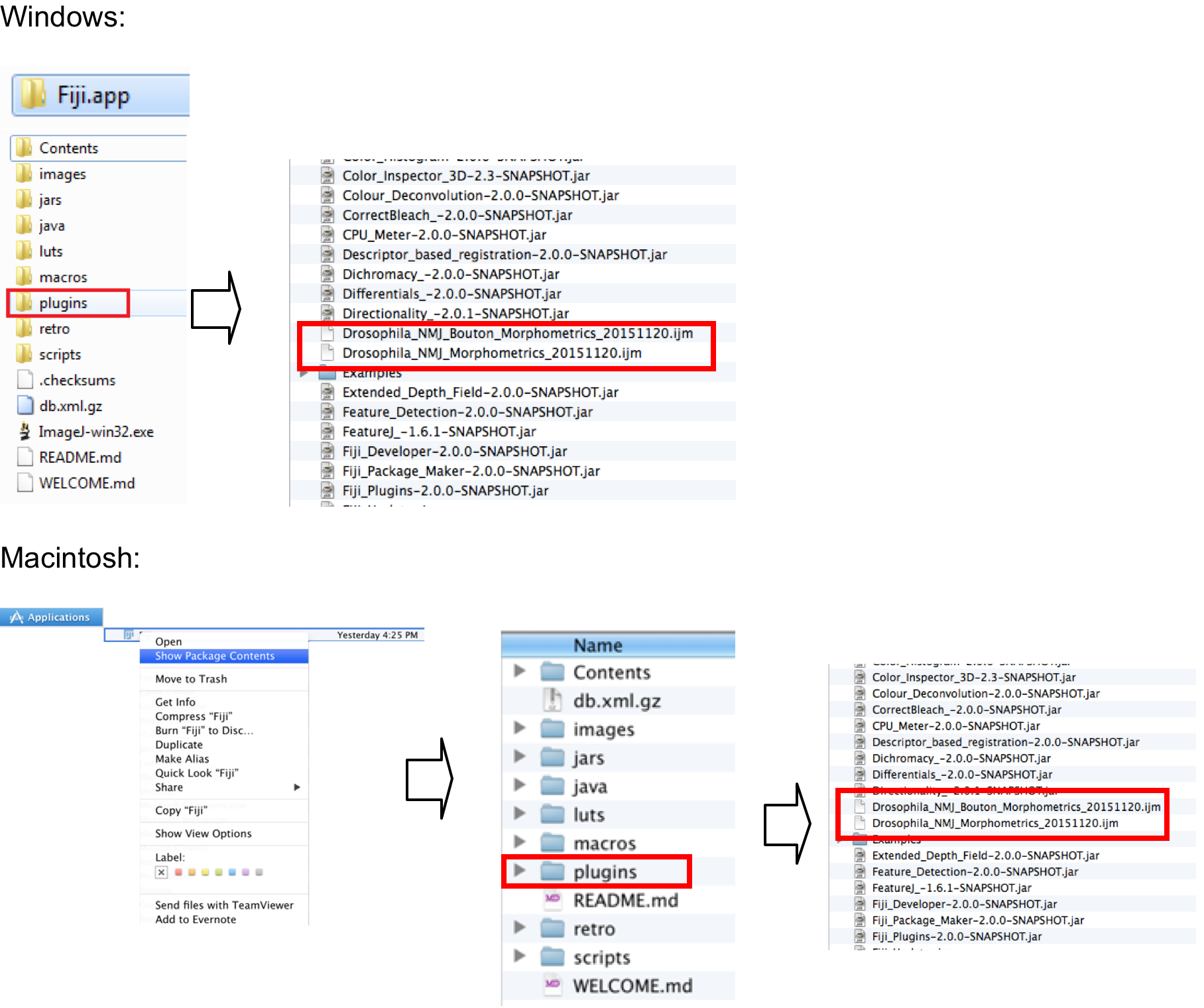


**■ Prepare your NMJ images**

**Preparation:**

- Two channel image stacks of the NMJ terminals need to be provided in order to run the macro. The first channel (channel 1) is used to create the outline and skeleton (based on Dlg1, Hrp ⇨‘Drosophila_NMJ_Morphometrics’, Syt or Csp-staining ⇨‘Drosophila_NMJ_Bouton_Morphometrics’) and the second channel (channel 2) is used to determine the number of Active Zones (Brp-staining). The channel order is important. If the channel order of the user images is not as described, follow section ‘change channel order’ on page 10 of this protocol.

**-** NMJ terminals can be imaged using a microscope of choice (fluorescence, confocal...). A two channel image stack of the desired NMJ should be taken, the number of planes of the stack can be as desired by the user, the stack should then be exported as individual ‘.tif files.

- File naming: Each imaged NMJ terminal should obtain a user-defined name, e.g. Synapse1. When the stacks are exported as separate .’.tif an individual file will be created for each channel and plane of the imaged stack. For example, if Synapse1 was imaged in 42 planes, 2x42 individual ’.tif files will be generated/stored. After the user-defined name ‘Synapse1’, additional information will be added by the microscope software in order to distinguish the generated images and allow relating them to each other. Frequently, this information will be added to the last part of the file name and will containing the z-plane and channel number. In our case, the first plane is defined as z-plane (z00) and the channel (ch00). Thus, the first plane of our stack ‘Synapse1’ will be stored with the following name:

“Synapse1_z00_ch00.tif” containing the Dlg1 channel and “Synapse1_z00_ch01.tif” for containing Brp channel; the second plane will follow as “Synapse1_z01_ch00.tif”, “Synapse1_z01_ch01.tif”, and successively till z042.

**If the images do not follow these criteria:**

If the user images do not follow these criteria, the user needs to perform a few manual steps in order the create images in the required format. The following interventions might be needed:

1. Images are not stored as a ‘.tif separate files’ (How to deal with images that are not stores as a ‘.tif separated files’, page 9.
2. The channels do not follow the required order (Change channel order, page 10)
3. The NMJ stack has more than 2 channels (Change channel order, page 10)

**■ Start the graphical interface**

1. Start Fiji.

For Macintosh users, it might be necessary to open Fiji with a control click, if

it is recognized as an ‘unidentified developer’.

2. Go to the Plugins tab and select the macro ‘Drosophila*_*NMJ_Morphometrics’ or ‘Drosophila_NMJ_Bouton_Morphometrics’ to open the graphical interface.

**
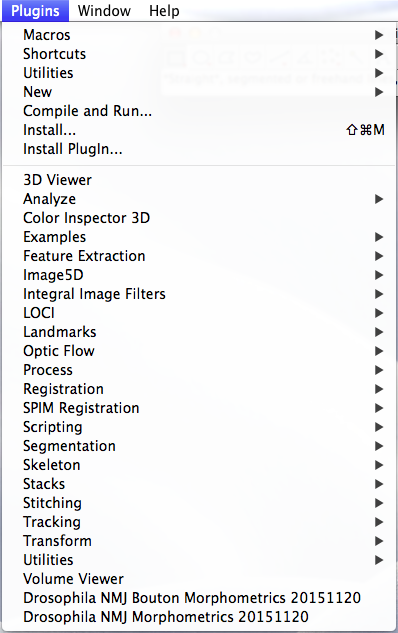
**

Note: After the macro installation, Fiji needs to be restarted in order to visualize the newly installed macros in the plugins folder.

**■ Sub macros**

The macro consists of three independent sub macros (‘Convert to stack’, ‘Define ROI’, and ‘Analyze’), which can be used separately or run in a consecutive manner. The user can check the box of the sub macros that he/she desires to run at the time in the graphical interface (see page 6).

*
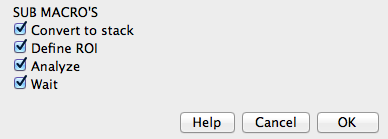
*

- Wait: Please be aware that, depending on your pc capacity, the sub macro ‘Analyze’ requires high capacity to run. The ‘wait’ checkbox allows the user to slow down the macro when counting Brp-positive spots to prevent incomplete counts when pc’s with lower capacity are used. We strongly recommend to always activate the ‘Wait’ checkbox when running the sub macro ‘Analyze’.

*Commentary:*

*‘Define ROI’ (Region Of Interest) requires manual input (page 11). We therefore recommend to run this sub macro separately when aiming to process a large amount of NMJ images. In this way, the ‘Analyze’ sub macro (page 13), which is time consuming, can process all images in succession without any user input.*

■ **Choose settings in the graphical interface**

The graphical interface displays the default settings of the macro, which can be adjusted according to the user needs. All settings are briefly summarized below. The ‘Help’ button provides further explanations.


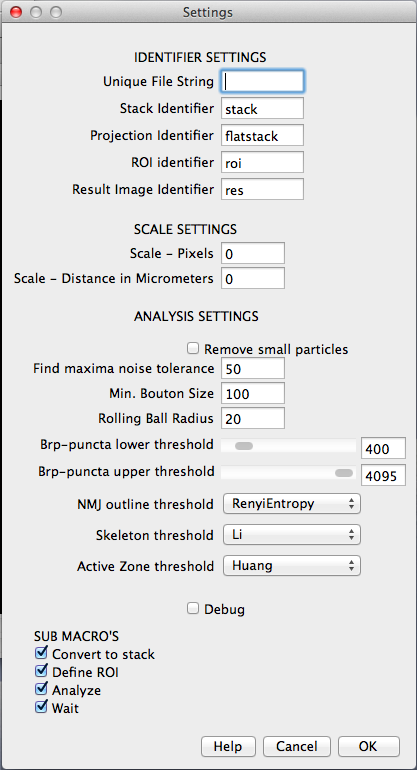
**1. Identifier settings**

- Unique File String: Is the generic repre-sentation of the last part of an image name, that uniquely identifies the first image of a image stack stored as a ‘.tif separated files. A more detailed description is given on page 8 of this protocol.

- Identifiers: The three sub macros generate in total four output files per NMJ image (stack, projection, ROI and result image). The identifier is added to the first part of the original file name to specify the created file type.

**2. Scale settings**

Here, the user has to set the scale for his/her images for the appropriate generation of quantitative results, e.g. if 1 pixels in the image corresponds to 2.5 micrometers, the user should fill Scale-Pixels=1, Scale-Distance in Micrometers=2.5. In case the entry is left at 0,0 the features: area, perimeter, length and longest branch length will be expressed in number of pixels.

**3. Analysis settings**

- Remove small particles: if this box is checked, particles smaller than 100 pixels in the macro ‘Drosophila_NMJ_Morphometrics’ and 10 pixels in ‘Drosophila_NMJ_Bouton_Morphometrics’ present in the first channel, are excluded from the analysis. This option might be used to filter out background noise. This option should always be activated when running Drosophila_NMJ_Bouton_Morphometrics.

- Rolling ball radius: In the first step of the macro, images are filtered applying a rolling ball background subtraction algorithm with a radius of (on default) 20 pixels.

- Minimum Bouton Size: Particles with a lower pixel area size (on default 100 in ‘Drosophila_NMJ_Morphometrics’ and 10 in ‘Drosophila_NMJ_Bouton_Morphometrics’) are not considered as objects of interest and are therefore excluded from the analysis.

- Find maxima noise tolerance: Brp-positive spots are counted by determining local maxima. Maxima are ignored if they do not stand out from the surroundings by more than this value.

- Brp-spots lower and upper threshold: Brp-positive spots are counted by determining local maxima. The observed maxima are thresholded. These numbers indicate the threshold range.

- NMJ outline threshold: The outline of the entire NMJ is defined by an auto-threshold selection based on Renyi’s Entropy algorithm in ‘Drosophila_NMJ_Morphometrics’, applied to the first channel as a default setting. This algorithm was shown to perform better than several other entropy-based threshold selection methods (see Methods section ‘Macro image analysis’ and its reference Sahoo P., *et al*. 1997 [64]). For ‘Drosophila_NMJ_Bouton_Morphometrics’ the most suitable algorithm to auto-threshold the images was found to be Moments, and is thus defined as the default auto-threshold for this macro.

- Skeleton threshold: The skeleton is a one-pixel thick axis along the center of the NMJ. Since Renyi’s Entropy algorithm sometimes performed too restrictive for this purpose, we set the less restrictive entropy-based auto-threshold Li’s Minimum Cross Entropy algorithm as default.

- Active zone threshold: Brp-positive spots were determined by using Huang’s fuzzy thresholding method, an auto-threshold that uses Shannon’s entropy function.

*Commentary:*

When using the macro for first time, it is required for an optimal output that the user explores the different auto-threshold algorithms and defines/choses the most suitable algorithm for its images. It is important to note that the users will likely need to play with several standard settings in order to adapt the macro to their images.

Fiji provides a plugin that offers various global (histogram-derived) thresholding methods. To determine the most suitable auto-threshold algorism to be used open 1 representative image and run Image>>Adjust>>AutoThreshold>>Try all, a binary thresholded result image with appear with all different auto-threshold algorithms available in Fiji, the user can then visually chose the more suitable algorithm for its images and change it accordingly in the macro settings. Please see <http://fiji.sc/Auto_Threshold> for more information.

The graphical interface of ‘Drosophila_NMJ_Morphometrics’ is set on the default settings used throughout this study. The drop-down menu allows the user to switch auto-thresholds.

We recommend more permissive thresholds to determine the NMJ skeleton and active zones, and more restrictive thresholds for NMJ area. An even more restrictive threshold should be used to define the NMJ area in the macro ‘Drosophila_NMJ_Bouton_Morphometrics’ for a proper detection of the boutons. The use of more permissive thresholds will lead to mistakes in bouton segmentation.

■ **Run sub macro ‘Convert to stack’**

**Important:**

The ‘Convert to stack’ sub macro only accepts input images stored in the following format:

1. **Images stored as ‘.tif separated files.** (‘.tif separated files are image stacks that are saved as individual ‘.tif files for each channel and plane).

2. **Images should contain 2 channels.**

For more information see section: ‘Prepare your images’,page 4 of this protocol.

**Procedure:**

1. Start the graphical interface of ‘Drosophila_NMJ_Morphometrics’ (page 5 of this protocol).

2. First, the user needs to fill the ‘Unique File String’ setting which is the identification signature that microscope softwares use to organize the different planes and channels when storing stacks as a separate ‘.tif files. Following our example (page 4) for our stack called Synapse1 separate ‘.tif files will be stored as follows:

Synapse1_z00_ch00.tif

Synapse1_z00_ch01.tif

Synapse1_z01_ch00.tif

Synapse1_z01_ch01.tif

…

Synapse1_z42_ch00.tif

Synapse1_z42_ch01.tif

The unique file string setting should be filled with: z00_ch00.tif, as highlighted in red. The entered string needs to specify the first plane of the first channel (lowest plane and channel number).

3. Deselect the checkboxes ‘Define ROI’, ‘Analyze’ and ‘Wait’ (if you only desire to run the ‘Convert to stack’ sub macro).

4. Optional: change the Stack and Projection identifiers (page 6 of this protocol).

‘Unique File String’, Stack Identifier’ and ‘Projection Identifier’ are the only 3 settings that may need to be changed when running ‘Convert to stack’ sub macro. Other settings options concern other parts of the macro..

5. Press ‘OK’ and a new window will open; ‘Choose a Directory’.

6. Select your main directory. Note that the macro is able to recognize files stored in subfolders of the main directory; it is thus possible to create several folders for your NMJ images (for example organized per genotype).

7. After pressing ‘Select’ the sub macro ‘Convert to stack’ automatically searches all folders (if any) within the selected main direction. After detecting all individual ‘.tif files, the macro converts these into a 2 channel hyperstack andits maximum intensity projection two newly created files are saved in the same directory as the individual files. Depending on the Stack and Projection Identifiers, these new files are named “stack_original name” for the newly produced hyperstack and “flatstack_original name”, for the maximum projection. E.g.:


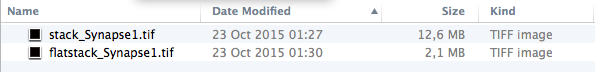


Note: do not run the ‘convert to stack’ sub macro repeatedly on the same separate ‘.tif files without removing the previously generated stack and flatstack files from the data folder. Repeated runs will generate files that contain multiple stacks, which can lead to Fiji ‘out of memory’ errors and to erroneous results.

■ **How to deal with images that are not stores as a ‘.tif separated files**

If images are not stored as a ‘.tif separated files, the user needs to manually perform the steps that are performed by the ‘Convert to stack’ sub macro.

1. Create a two channel hyperstack where channel 1 belongs to Dlg1 (or Hrp, Csp or Syt-) staining and channel 2 to Brp staining. Name the file: ‘stack_original_name.tif’ and save it as ‘.tif file. More information on how to create hyperstacks can be found at: <http://rsbweb.nih.gov/ij/docs/guide/146-8.html>.

For Fiji supported formats, when images are imported to Fiji, an interface will automatically appear where the user can readily chose to open the stored image as hyperstack. In this case, the user needs to open the file and save the hyperstack as ‘.tif with the name stack_original_name.tif. (Please be aware that the channel order of the hyperstack may need to be changed before saving the hyperstack, see page 10 of this protocol). For more information see example 3 in the macro repository.


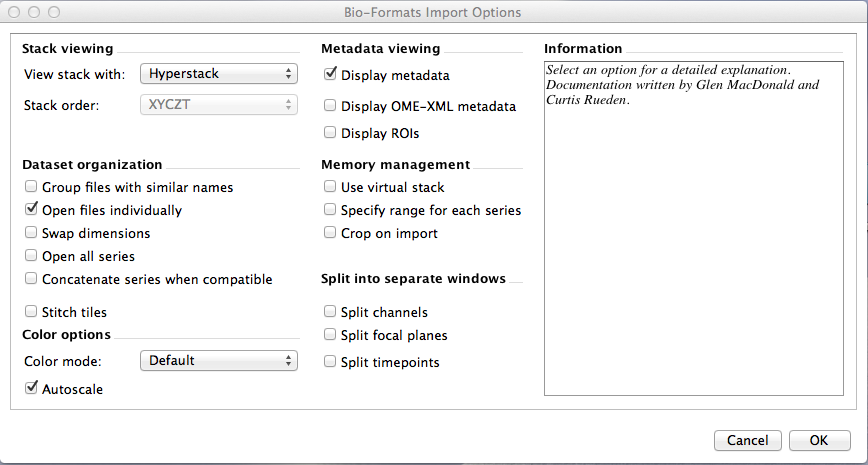


2. Create a maximum projection of the generated hyperstack and provide the following name: ‘flatstack_original_name.tif’ and save it as a ‘.tif, following the commands:

Fiji menu: Image>>Stack>>Z Project…>>Max Intensity


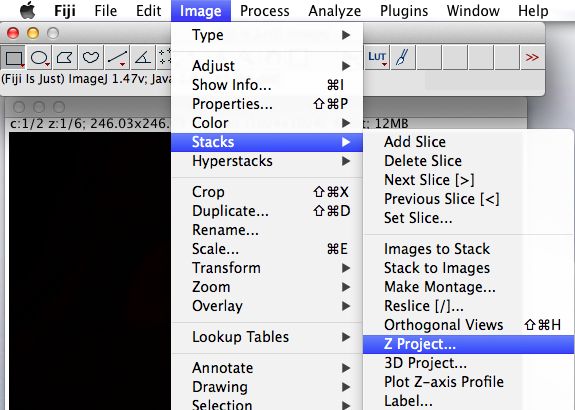


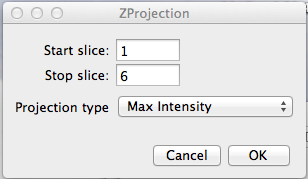


Both files (‘stack_original_name.tif’ and ‘flatstack_original_name.tif’) need to be saved in the same folder. Once these steps are performed, either by the submacro ‘Convert to Stack’ or manually, the ‘Define ROI’ and ‘Analyze’ sub macros will run without problems.

■ **Change channel order:**

The first channel should correspond to Dlg1 (or alternatively Hrp, Csp or Syt-staining). The second channel should correspond to Brp. In case the channel order is inverted, the macro will run, however, the wrong thresholds will be applied to each channel, producing non-sense results.

1. To invert the channel order of a hyperstack, please follow:


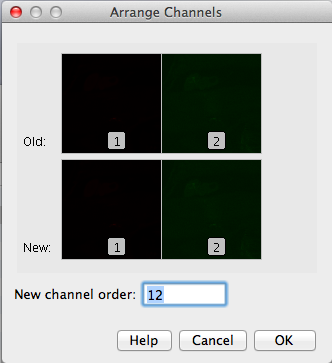
FIJI menu: Image>>Arrange Channels>> New channel order >>Type channel order as desired in the white box.


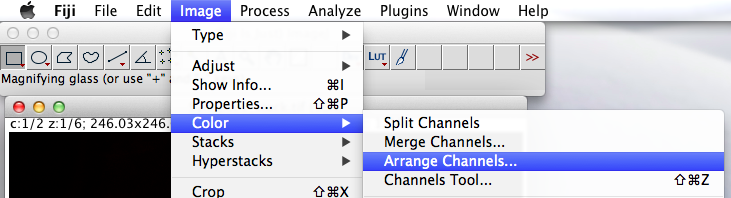


Note: If the separated ‘.tif files have a inverted channel order, we recommend to the user to run the sub macro ‘Convert to stack’, and then manually change the channel order of the newly created stack files.

2. The image should contain two channels. In case the user image contains three or more channels, please reduce the hyperstack dimension by: Image>>Arrange Channels>> New channel order >>Type in the 2 channel names that have to be analyzed in the correct order into the white box.

**■ Run sub macro ‘Define ROI’**

This sub macro will automatically open all maximum projection images labeled (as default) as flatstack_original_name. In these images the user needs to manually define the Region Of Interest ROI (surrounding the NMJ terminal to be quantified). This step is required to exclude the evaluation of parts of other terminals that might be contained in the images, see below). The output of this sub macro will be a binary image where the region of interest is represented in white on black background. The ROI image created is stored under the name (as default) roi_original_name.tif in the same directory as the original processed images.

1. Start the graphical interface of ‘Drosophila_NMJ_Morphometrics’ (page 5 of this protocol).

2. Deselect the checkboxes ‘Convert to Stack’, ‘Analysis’ and ‘Wait’ when running ‘Define ROI’ independently (we here focus on creating regions of interest).

3. Optional: change the ROI identifier (page 6 of this protocol). This sub macro is only dependent of ‘ROI identifier’ and ‘Projection identifier’ settings; all other settings can be neglected in case of running only this sub macro.

4. Press ‘OK’: a new window will open ‘Choose a Directory’.

5. Select your main directory. Note that the macro is able to recognize subfolders in the main directory; it is possible to create several folders for your NMJ images (for example ordered per genotype).

6. After pressing ‘Select’ the sub macro ‘Define ROI’ automatically searches through all folders (if any) within the selected main direction. After detecting all suitable files (in this case pairs of stacks and projections), the macro progresses each individual file separately. The first projection will appear.

7. Select ‘Freehand selections’ (indicated by the red square in the example below) and draw a region that exclusively includes the complete NMJ terminal of interest (and no other types of NMJ terminals). Click ‘OK’ in the window ‘Define terminal’. The macro will continue with the next projection.


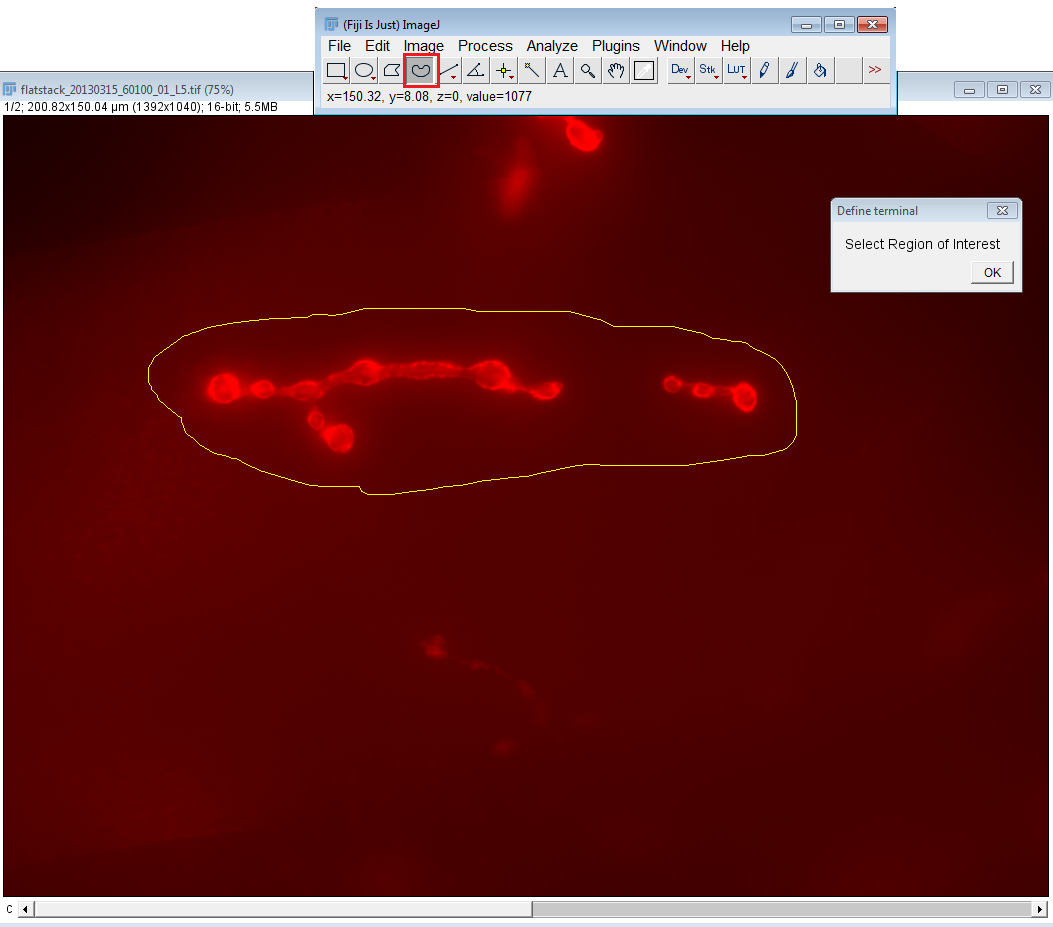


8. Depending on the ROI Identifier, the ROI file is named “roi_original name” and is saved in the same directory as the stack and projection. It consists of a binary mask.


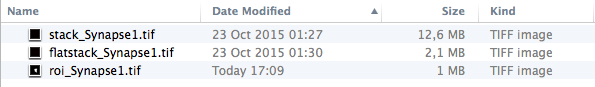


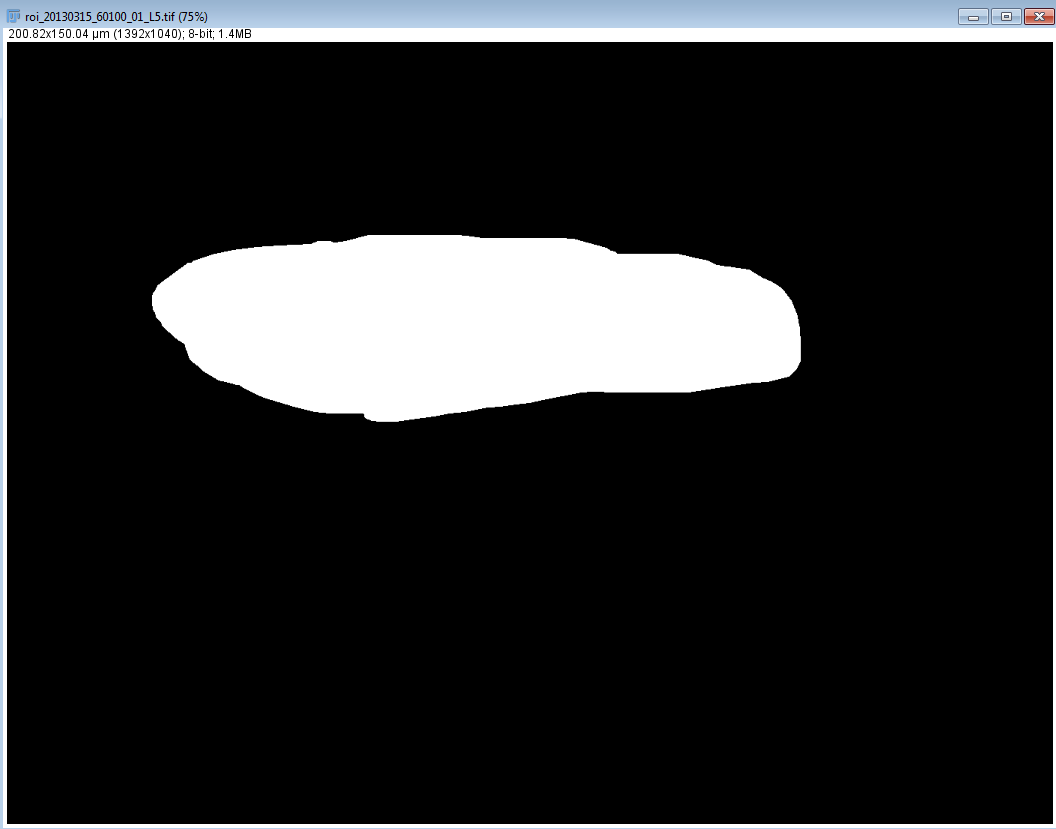


**■ Run sub macro ‘Analyze’**

This sub macro will quantify the 9 different parameters of the NMJ terminal contained in the Region Of Interest (ROI) selected by the user, and will create i) a result output file (results.txt) with the corresponding measurements and ii) a result image (res_original_name.tif) where the different applied thresholds and measurements by the macro are visualized. Since with the user-defined macro settings, there will always be a small percentage of images where the macro will not perform adequately, we recommend to inspect all result images to detect unsatisfying segmentation and exclude those pictures from the analysis (adjusting the macro settings to properly segment 100% of the images can be a very laborious procedure). The fraction of properly segmented images in our hands has always been between 90-95%, but it depends on the quality of the immunostainings and microscopy technique. Evidently, if the quality of the images is low, the macro will generate higher ratios of unsatisfactory measurements.

1. Start the graphical interface of ‘Drosophila_NMJ_Morphometrics’ (page 6-7 of this protocol).

2. Deselect the checkboxes ‘Convert to Stack’, and ‘Define ROI’ (We here focus on the macro analysis).

3. Optional: change the Result Image Identifier (page 6 of this protocol).

4. Change the scale settings according to your images (page 6 of this protocol).

5. Change the Analysis settings of the graphical interface (page 6-7 of this protocol for detailed information).

6. Press ‘OK’: a new window will open ‘Choose a Directory’.

7. Select your main directory. Note that the macro is able to recognize subfolders in the main directory; it is possible to create several folders for your NMJ images (for example organized per genotype).

8. After Pressing ‘Select’ the sub macro ‘Analyze’ automatically searches all folders (if any) within the selected main direction. After detecting all suitable files (in this case the match of a stack, projection and ROI file), the macro processes each image individually and sequentially. This can take up to 8 minutes (depending on your pc capacity) per stack. During analysis, the ROI defines the region in which the stack file is analyzed.

*Commentary:*

*- It is highly recommended not to run other software while this macro is running.*

*- The macro determines the outline and skeleton in the first channel of the stack and Brp-positive spots are identified in the second channel of the stack. The Fiji option ‘Arrange channels’ allows the user to switch channels, if necessary (Image 🡪 Color 🡪 Arrange channels).*

9. Depending on the Result Image Identifier, the Result file is named “res_original name” and is saved in the same directory as the stack, projection, and ROI files.


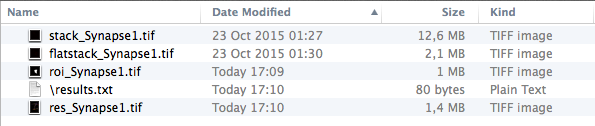


It consists of a small stack (containing 3 images). The first image highlights the outline and skeleton on a first channel image (Dlg1 in case of the example below). The second image is a copy of the first image, and additionally shows the identified Brp-positive spots as schematized foci. The third image provides the maximum projection of the second channel.


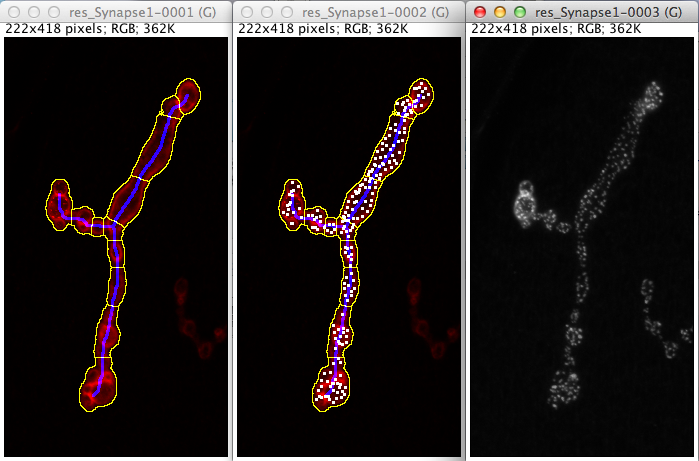


10. The quantitative results of all parameters and images that were progressed by the macro are combined and listed in the ‘results.txt’ output file. The results are linked to the file name and the parameters are summarized after the image name in the following order:

**Drosophila_NMJ_Morphometrics (Dlg1 or Hrp and Brp labeling):**

| **Parameter** | **NMJ structure** | **Explanation** |
| --- | --- | --- |
| Area (µm^2^) | NMJ outline | The surface of the complete labeled NMJ |
| Perimeter (µm) | NMJ outline | The perimeter belonging to the area |
| #Boutons | NMJ outline | The number of periodic enlargements of the NMJ |
| Length (µm) | Skeleton | The total length of the complete NMJ |
| Longest branch length (µm) | Skeleton | The sum of the length of the longest continuous path connecting any two end points of the NMJ |
| #Branches | Skeleton | The total number of branches |
| #Branching points | Skeleton | The number of branching points |
| #Islands | Skeleton | The number of Dlg1-staining compartments (or any other staining) |
| #Active zones | Brp-positive spots | The number of active zones, based on Brp staining |

**Drosophila_NMJ_Bouton_Morphometrics (Syt1 or Csp and Brp labeling):**

| **Parameter** | **NMJ structure** | **Explanation** |
| --- | --- | --- |
| Boutons | NMJ outline | The number of periodic enlargements of the NMJ |
| Bouton area | NMJ outline | The surface of all boutons |
| Length (µm) | Skeleton | The total length of the complete NMJ |
| Longest branch length (µm) | Skeleton | The sum of the length of the longest continuous path connecting any two end points of the NMJ |
| #Branches | Skeleton | The total number of branches |
| #Branching points | Skeleton | The number of branching points |
| #Islands | Skeleton | The number of Dlg1-staining compartments (or any other staining) |
| #Active zones | Brp-positive spots | The number of active zones, based on Brp staining |

■ **Settings used for this paper**

**Dlg1 – Brp (high content microscope) Dlg1 – Brp (Confocal)**

**
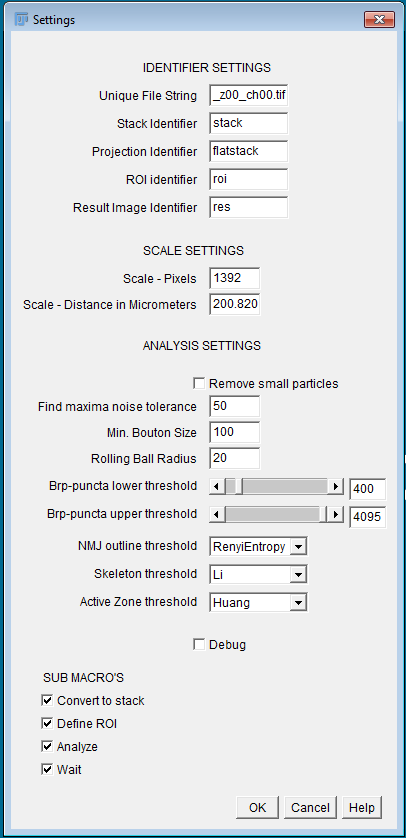

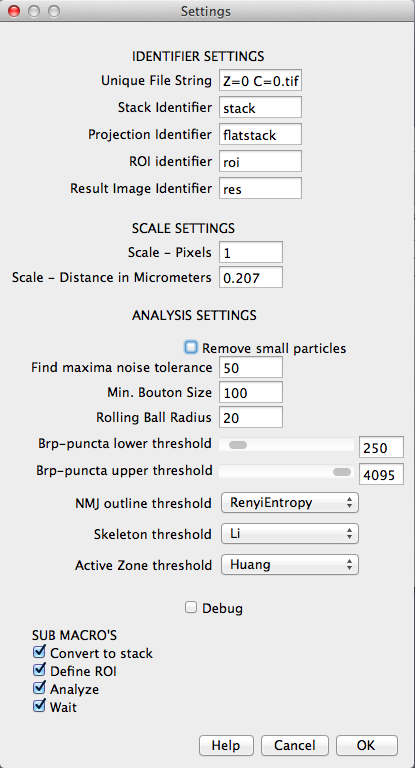
**

**
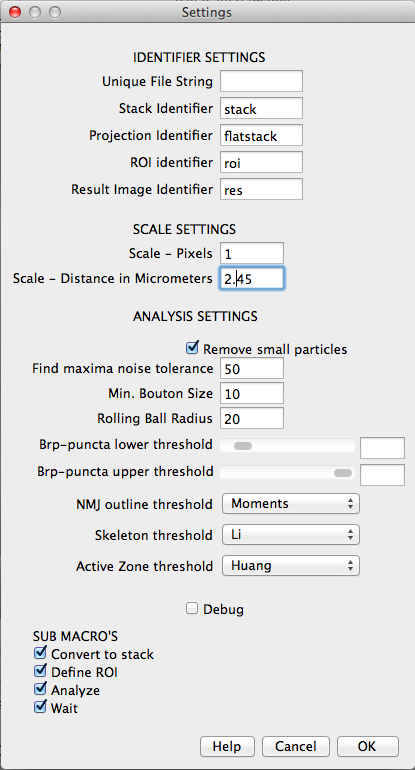
Hrp – Brp** **Syt– Brp**


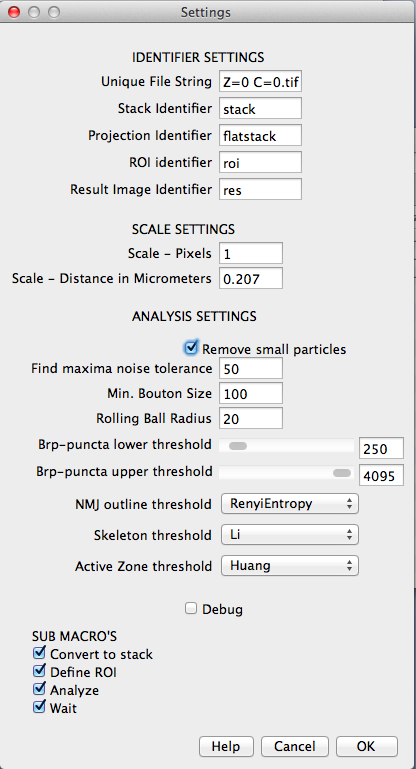

Supplement: S1 Protocol — (DOCX) [file pcbi.1004823.s010.docx]
